# CD38 as Immunotherapeutic Target in Light Chain Amyloidosis and Multiple Myeloma—Association With Molecular Entities, Risk, Survival, and Mechanisms of Upfront Resistance

**DOI:** 10.3389/fimmu.2018.01676

**Published:** 2018-07-20

**Authors:** Anja Seckinger, Jens Hillengass, Martina Emde, Susanne Beck, Christoph Kimmich, Tobias Dittrich, Michael Hundemer, Anna Jauch, Ute Hegenbart, Marc-Steffen Raab, Anthony D. Ho, Stefan Schönland, Dirk Hose

**Affiliations:** ^1^Medizinische Klinik V, Universitätsklinikum Heidelberg, Heidelberg, Germany; ^2^Institut für Humangenetik, Universität Heidelberg, Heidelberg, Germany

**Keywords:** multiple myeloma, amyloidosis, immunotherapy, CD38, alternative splicing, survival

## Abstract

Monoclonal antibodies against the cell surface antigen CD38, e.g., isatuximab, daratumumab, or Mor202, have entered the therapeutic armamentarium in multiple myeloma due to single agent overall response rates of 29 vs. 36 vs. 31%, effectivity in combination regimen, e.g., with lenalidomide or bortezomib plus dexamethasone, and tolerable side effects. Despite clinical use, many questions remain. In this manuscript, we address three of these: first, upfront CD38 target-expression in AL-amyloidosis, monoclonal gammopathy of unknown significance (MGUS), asymptomatic, symptomatic, and relapsed multiple myeloma. Second, relation of CD38-expression to survival, disease stages, molecular entities, and high-risk definitions. Third, alternative splicing or lack of CD38-expression as potential mechanisms of upfront resistance. We assessed CD138-purified plasma cell samples from 196 AL-amyloidosis, 62 MGUS, 259 asymptomatic, 764 symptomatic, and 90 relapsed myeloma patients, including longitudinal pairs of asymptomatic/symptomatic (*n* = 34) and symptomatic/relapsed myeloma (*n* = 57) regarding interphase fluorescence *in situ* hybridization (*n* = 1,380), CD38-expression by gene expression profiling (*n* = 1,371), RNA-sequencing (*n* = 593), and flow cytometry (*n* = 800). Samples of normal bone marrow plasma cells (*n* = 10), memory B-cells (*n* = 9), polyclonal plasmablastic cells (*n* = 9), and human myeloma cell lines (*n* = 54) were used as comparators. CD38 was expressed in all malignant plasma cell samples, but significantly lower compared to normal plasma cells with small but significant downregulation in longitudinal sample pairs. Higher CD38 expression was associated with the presence of t(4;14) and high-risk according to the UAMS70-gene score, lower expression was associated with del17p13 and hyperdiploidy in symptomatic myeloma as well as t(11;14) in asymptomatic myeloma. Higher CD38-expression was associated with slower progression to symptomatic and relapsed myeloma and better overall survival in the latter two entities. CD38 expression, t(4;14), del17p13, and gain of 1q21 are independently prognostic in multivariate analysis. By contrast, high CD38-expression is associated with adverse survival in AL-amyloidosis. Regarding mechanisms of upfront anti-CD38-treatment resistance, lack of CD38-expression and alternative splicing of receptor binding-sites could be excluded. Here, of the two protein coding CD38-transcripts CD38-001 (eight-exon, full length) and CD38-005 (truncated), CD38-001 conveyed >97% of reads spanning the respective CD38 splice junction.

## Introduction

Monoclonal antibodies against the cell surface antigen CD38, e.g., isatuximab, daratumumab, or Mor202, have entered the therapeutic armamentarium in multiple myeloma due to single agent overall response rates of 29 vs. 36 vs. 31%, respectively, effectivity in combination regimen, e.g., with lenalidomide or bortezomib plus dexamethasone, and tolerable side effects (1–6).

Despite clinical use, many questions remain. First, CD38-target expression has mainly been studied for either relapsed/refractory myeloma, small patient cohorts, or after gating using CD38 (among other markers) to identify the plasma cell population (7–11). The latter analysis implies that per definition (as gated) all plasma cells show expression of CD38, i.e., was not designed to address variation or absence thereof. What is its expression in a large patient cohort without these limitations? Is it higher expressed in previously untreated patients and especially asymptomatic stages, suggesting earlier use? Information is also unavailable for patients with AL-amyloidosis. Second, no data are available for the presence of CD38-expression in high-risk entities as defined by molecular alterations including fluorescence *in situ* hybridization or gene expression-based factors. For our German-Speaking-Myeloma-Multicenter-Group (GMMG), this posed a surprising hurdle in defining the rationale of our CONCEPT trial (EudraCT 2016-000432-17), adding anti-CD38 treatment for symptomatic *high-risk* patients. Likewise, whether CD38-expression is associated with prognosis. Third, whether upfront resistance to single-agent anti-CD38 treatment could be mediated by either the absence of CD38-expression or alternative CD38-splicing removing the antibody binding site in a subfraction of patients.

## Materials and Methods

### Patients, Healthy Donors, and Samples

**Figure 1:**
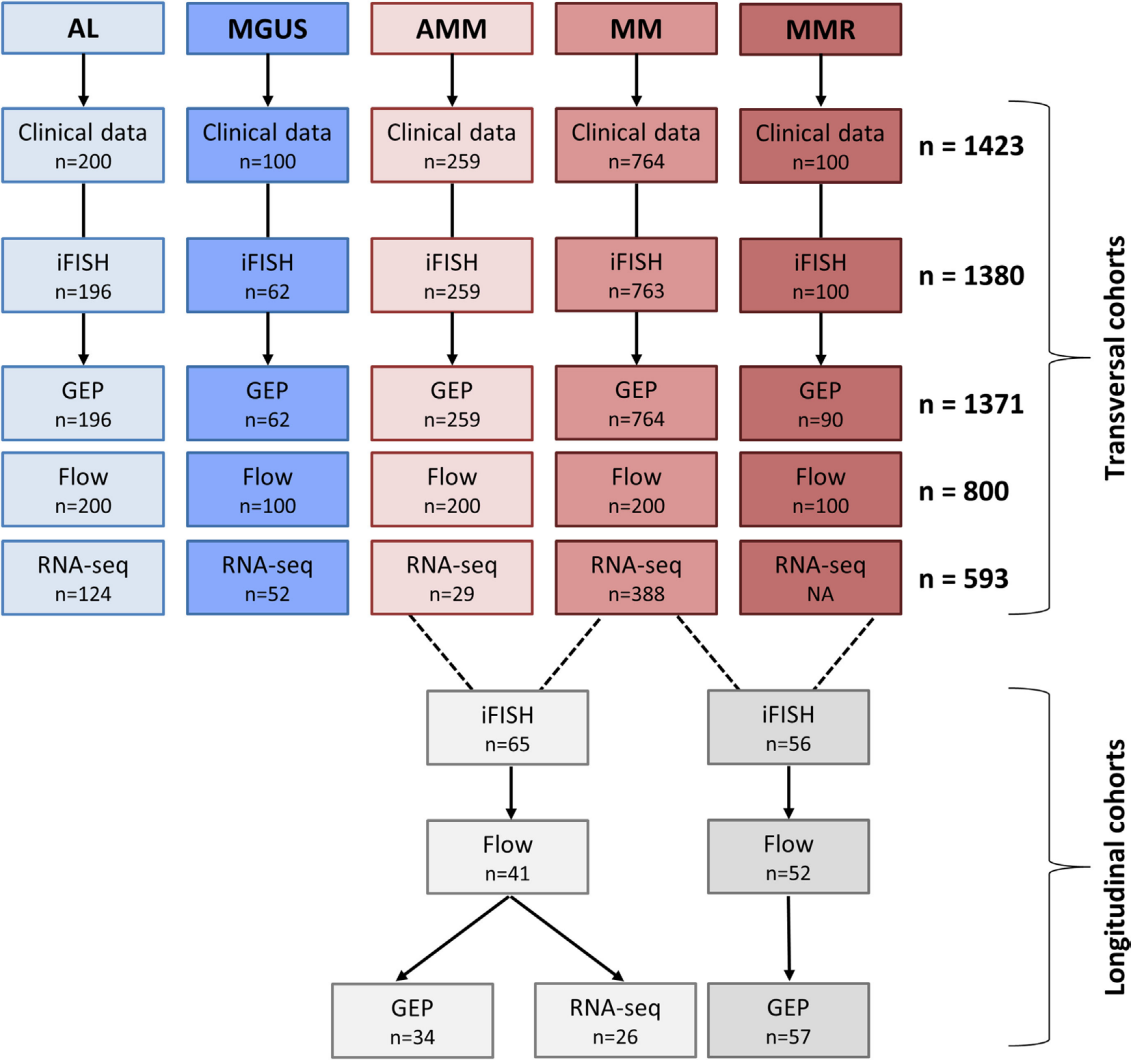
Overview of patient cohorts and investigations. Shown are the patient numbers within our transversal and longitudinal cohorts for whom the respective data were available. Abbreviations: AL, AL-amyloidosis; MGUS, monoclonal gammopathy of unknown significance; AMM, asymptomatic multiple myeloma; MM, therapy-requiring multiple myeloma; MMR, relapsed multiple myeloma; iFISH, interphase fluorescence *in situ* hybridization; GEP, gene expression profiling; RNA-seq, RNA-sequencing; Flow, flow cytometric analysis.

Consecutive patients with AL-amyloidosis (*n* = 196), monoclonal gammopathy of unknown significance (MGUS) (*n* = 62), asymptomatic (*n* = 259), symptomatic, therapy-requiring (*n* = 764), and relapsed/refractory myeloma (*n* = 90), as well as healthy donors (*n* = 19) as comparators were included in the study approved by the ethics committee (#229/2003, #S-152/2010) after written informed consent (Figure 1; Table S1 in Supplementary Material). Normal bone marrow plasma cells (BMPCs) and myeloma cells were purified using anti-CD138 microbeads (Miltenyi Biotec, Bergisch Gladbach, Germany) (12, 13). Peripheral blood CD27^+^ memory B-cells (MBCs) (*n* = 9) were FACS-sorted and polyclonal plasmablastic cells (PPCs) (*n* = 9) were *in vitro* differentiated as described (14).

The human myeloma cell lines (HMCLs) L363, SK-MM-2, LP-1, RPMI-8226, AMO-1, KMS-18, JIM-3, JJN3, KARPAS-620, KMS-12-BM, ANBL-6, KMS-11, MM1S, NCI-H929, KMS-12-PE, OPM-2, MOLP-8, MOLP-2, KMM-1, U266, and EJM were purchased from the German Collection of Microorganisms and Cell Cultures (Braunschweig, Germany), American Type Cell Culture (Wesel, Germany), or the National Institutes of Biomedical Innovation, Health and Nutrition (Osaka, Japan); the HG-lines HG1, HG3, HG4, HG5, HG6, HG7, HG8, HG9, HG11, HG12, HG13, HG14, HG15, HG17, and HG19 were generated at the Myeloma Research Laboratory Heidelberg, the XG1, XG2, XG3, XG4, XG5, XG6, XG7, XG10, XG11, XG12, XG13, XG14, XG16, XG19, XG20, XG21, XG22, XG23, and XG24 at Montpellier. Cell line identity was assessed for proprietary cell lines by DNA-fingerprinting, mycoplasma-contamination excluded by PCR-based assays, and EBV-infection status by clinical routine PCR-based diagnostics.

### Measurement of CD38-Expression

#### Gene Expression Profiling (GEP)

Gene expression profiling using U133 2.0 plus arrays (Affymetrix, Santa Clara, CA, USA) was performed as published (12, 13, 15). Expression data are deposited in ArrayExpress under accession numbers E-MTAB-4715, E-MTAB-4717, E-MTAB-5212, E-TABM-937, and E-TABM-1088.

#### RNA-Sequencing

RNA-sequencing was performed as published (12, 16, 17). In brief, full-length double-stranded cDNA was generated from 5 ng of total RNA and amplified using the SMARTer Ultra Low RNA Kit (Illumina, San Diego, CA, USA). Library preparation was performed from 10 ng of fragmented cDNA using the NEBNext Chip-Seq Library Prep protocol (New England BioLabs, Ipswich, MA, USA). Libraries were sequenced on an Illumina Hiseq2000 with 2 × 50-bp paired-end reads.

#### Flow Cytometry

Bone marrow aspirates were stained with anti-CD138-PE (clone B-B4, Miltenyi Biotec) and anti-CD38-FITC (clone HB 7, Becton Dickinson, Heidelberg, Germany). Cells stained with corresponding isotype antibodies were used as control. Analysis was performed on a FACSCalibur (Becton Dickinson) using BD CellQuest software (Becton Dickinson) and FlowJo version x.0.7 (FLOWJO, LLC, Ashland, OR, USA).

### Interphase Fluorescence *In Situ* Hybridization (iFISH)

Interphase fluorescence *in situ* hybridization analysis was conducted on CD138-purified plasma cells using probes for chromosomes 1q21, 5p15, 5q31 or 5q35, 8p21, 9q34, 11q22.3 or 11q23, 13q14.3, 15q22, 17p13, 19q13, IgH-breakapart, as well as translocations t(4;14) (p16.3;q32.3), t(11;14) (q13;q32.3), and t(14;16) (q32.3;q32) (Kreatech, Amsterdam, The Netherlands and MetaSystems, Altlussheim, Germany) as published (18, 19).

### Statistical Analysis

Microarray gene expression analyses were performed on GC-RMA (20) preprocessed data for our cohort as well as mas5 for the total therapy 2 and 3 cohort (see below). Due to two different IVT labeling kits used, batch correction was performed using ComBat (21). To assess presence or absence of gene expression, the “Presence-Absence calls with Negative Probesets” (PANP) algorithm (22) was used. The EMC92-gene score (23), UAMS70-gene score (24), Rs-score (25), our gene expression-based proliferation index (GPI) (13), as well as the TC (26) and the molecular classification (24) have been calculated as published. For calculation of the UAMS70-score and the TC-classification, data were normalized using mas5.

Next generation sequencing RNA fastq-files were analyzed with STAR with default options (27). A genome reference was generated with STAR using GRCh38 genome and annotation information of Ensembl databases release 82 (28). Files were aligned to GRCh38 genome build and reads counted per gene and splice junction. STAR uses HTSeq (29) internally for counting reads and sorts the resulting bam files by coordinate. Technical replicated were summed and reads per gene were normalized with edgeR (30). Therefore, normalization factors were calculated with default options and counts per million were computed with accounting for library size. Then log2 transformation was performed with a prior count of 1. A transcript reference was generated with RSEM using GRCh38 genome and annotation information of Ensembl databases release 82 and using option–star for creating STAR indices. Files were aligned with STAR per transcripts and expression was calculated with RSEM (31) with default options. Plots were generated with RSEM function “rsem-plot-transcript-wiggles” presenting read depth of uniquely and multiple mapping reads.

Expression values from flow cytometry were analyzed as log transformed MFI values.

Differences in clinical parameters [e.g., ISS stage (32), tumor mass surrogates, i.e., free light chain ratio involved:uninvolved of ≥100 (33), immunoparesis as defined by suppression of one or two of the non-involved immunoglobulins below the lower limit of normal (34), model according to Kyle et al. (35) and the Mayo-model (36), number of affected organs, or the Mayo2004-score (37)], cytogenetics and between defined groups were investigated by exact Wilcoxon rank-sum test (38).

Computations were performed using R^1^ version 3.3 and Bioconductor (39), version 3.3.^2^ Effects were considered statistically significant if the *p* value of corresponding statistical tests was below 5%. To correct for multiple testing, *p* values in Table 3 were adjusted with Benjamini–Hochberg correction (40).

Overall and event-free survival was investigated for AL-amyloidosis, symptomatic multiple myeloma undergoing high-dose therapy, and refractory multiple myeloma, and progression-free survival for asymptomatic myeloma patients using Cox’s proportional hazard model (41). In AL-amyloidosis, hematologic event-free survival, i.e., hematologic relapse or progression, initiation of a new therapy or death (whichever came first), were defined as events (42). Survival data were validated by an independent cohort of 701 myeloma patients treated with high-dose chemotherapy within the total therapy 2 or 3 protocol (TT2/TT3), respectively (43, 44).

For the analysis of the prognostic value of CD38, patients with “high” and “low” CD38-expression in microarray data were delineated using maximally selected rank statistics^3^ with thresholds calculated for each disease entity. For validation purposes, thresholds calculated on our cohort of symptomatic myeloma patients were applied to the TT2/TT3 cohort.

Survival curves were computed with nonparametric survival estimates for censored data using the Kaplan–Meier method (45). Difference between the curves was tested using the G-rho Log-rank test (46).

## Results

### Expression of CD38 in Malignant Plasma Cells

**Figure 2:**
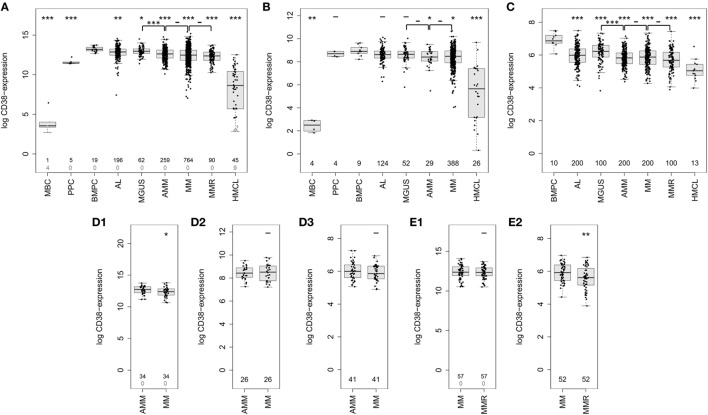
Expression of CD38 in normal and malignant plasma cells. CD38-expression determined by **(A)** gene expression profiling using Affymetrix U133 2.0 DNA-microarrays (probe set 205692_s_at) in normal plasma cell precursors, i.e., memory B-cells (MBCs, *n* = 5), *in vitro* generated polyclonal plasmablastic cells (PPC, *n* = 5), and normal bone marrow plasma cells (BMPCs, *n* = 19), malignant plasma cells from patients with AL-amyloidosis (AL, *n* = 196), monoclonal gammopathy of unknown significance (MGUS, *n* = 62), asymptomatic myeloma (AMM, *n* = 259), symptomatic myeloma (MM, *n* = 764), relapsed multiple myeloma (MMR, *n* = 90), as well as human myeloma cell lines (HMCLs, *n* = 54). Black numbers correspond to the number of samples which express CD38 according to the PANP algorithm, gray numbers reflect those that do not express CD38. **(B)** Confirmation using RNA-sequencing of PPC (*n* = 4), MBC (*n* = 4), normal (*n* = 9) and malignant plasma cells from patients with AL (*n* = 124), MGUS (*n* = 52), AMM (*n* = 29), and MM (*n* = 388), as well as cell lines (*n* = 26). **(C)** CD38 expression on protein level as determined by flow cytometry on the surface of normal (*n* = 10) and malignant plasma cell samples from patients with AL-amyloidosis (*n* = 200), MGUS (*n* = 100), AMM (*n* = 200), MM (*n* = 200), and MMR (*n* = 100). **(D)** Expression of CD38 in longitudinal (paired) samples of asymptomatic and symptomatic myeloma as per **(D1)** gene expression profiling (GEP) (*n* = 34 pairs, *p* < 0.05), **(D2)** RNA-sequencing (*n* = 26 pairs), and **(D3)** flow cytometry (*n* = 41 pairs). **(E)** Expression of CD38 in longitudinal samples of symptomatic and relapsed myeloma according to **(E1)** GEP (*n* = 57 pairs, *p* = n.s.) and **(E2)** flow cytometry (*n* = 52 pairs, *p* < 0.01). Significant difference between the groups is depicted by one asterisk (*) for a level of *p* < 0.05, two asterisks (**) for a level of *p* < 0.01, and three (***) for *p* < 0.001 with corresponding patient numbers being depicted in the boxplots. In the upper row, asterisks compare the expression of cohorts against the expression of BMPCs, the lower row compares disease entities pairwise.

Using GEP, we found CD38 expressed in all 1,371 malignant plasma cell samples derived from symptomatic, relapsed, asymptomatic, MGUS-, and AL-amyloidosis patients (Figure 2A; Table 1). Expression of CD38 was validated by RNA-sequencing (*n* = 593) and flow cytometry (*n* = 800) (Figures 2B,C; Table 1). The expression levels in all entities were significantly lower compared to normal BMPCs, but remained for all samples above the level of MBCs, in which CD38-expression is absent. Expression levels varied within each of the entities by two orders of magnitude (Table 1). In paired samples, a trend for lower expression in more advanced stages was found. This difference was significant for CD38-expression (GEP) in asymptomatic vs. symptomatic myeloma (*n* = 34, *p* = 0.01; Figures 2D1–D3) and symptomatic vs. relapsed myeloma in flow cytometry (*n* = 52, *p* = 0.001), however, the absolute difference was small (Figures 2E1,E2). The same trend can also be seen for the subgroup of patients with AL-amyloidosis: lower CD38-expression is found with underlying asymptomatic myeloma compared to MGUS, being significant for GEP (*p* = 0.02) and flow cytometry (*p* = 0.008), respectively.

**Table 1:** Expression (log) of CD38 by gene expression profiling (GEP, RNA-sequencing) and flow cytometry.

Population	GEP						RNA-Seq						Flow cytometry			
	CD38 median	CD38 SD	CD38 min.	CD38 max.	FC MBC	LFC MBC	CD38 median	CD38 SD	CD38 min.	CD38 max.	FC MBC	LFC MBC	CD38 median	CD38 SD	CD38 min.	CD38 max.
MBC	3.56	1.43	2.70	6.44	–	–	2.50	0.54	1.83	2.92	–	–	–	–	–	–
PPC	11.47	0.35	11.40	12.24	241	229	8.70	0.25	8.43	8.90	74	61	–	–	–	–
BMPC	13.20	0.31	12.68	13.75	800	558	8.90	0.46	8.22	9.64	85	53	6.87	0.42	6.07	7.49
AL	12.88	0.82	7.43	14.57	641	15	8.62	0.61	6.30	10.12	70	14	5.99	0.60	4.09	7.49
MGUS	12.97	0.56	11.40	14.52	681	229	8.65	0.68	5.80	10.04	71	10	6.23	0.60	3.83	7.33
AMM	12.63	0.80	10.10	14.78	538	93	8.41	0.79	5.48	9.52	60	8	5.83	0.51	4.38	7.15
MM	12.48	1.04	7.05	15.42	484	11	8.47	0.90	4.05	10.69	63	3	5.88	0.60	4.28	7.34
MMR	12.38	0.81	10.27	13.75	252	105	–	–	–	–	–	–	5.68	0.67	3.89	7.44
HMCL	8.64	3.01	2.84	12.53	34	0.98	5.65	2.42	0.31	9.68	9	1	5.04	0.64	3.99	6.52

*MBC, memory B-cells; PPC, polycloncal plasmablastic cells; BMPC, (normal) bone marrow plasma cells; AL, AL-amyloidosis; MGUS, monoclonal gammopathy of unknown significance; (A)MM, (asymptomatic) multiple myeloma; MMR, relapsed multiple myeloma; HMCL, human myeloma cell line; SD, standard deviation; Min, minimum expression; Max, maximum expression; FC MBC, fold change of CD38 expression of the respective population vs. MBC; LFC MBC, lowest fold change of CD38 expression of the respective population vs. MBC*.

### Relation of CD38-Expression to Survival, Molecular Entities, and High-Risk Definitions

**Figure 3:**
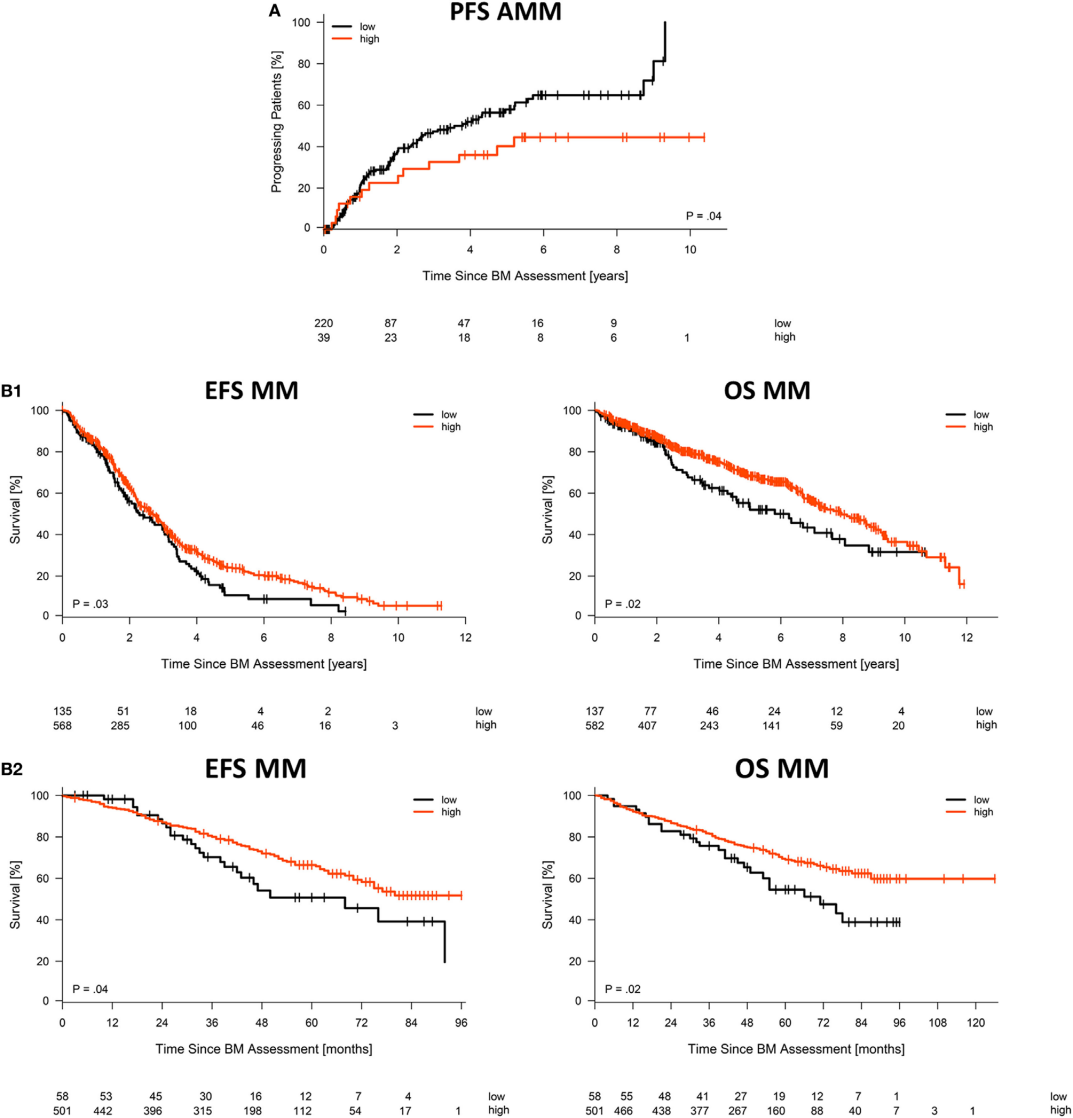

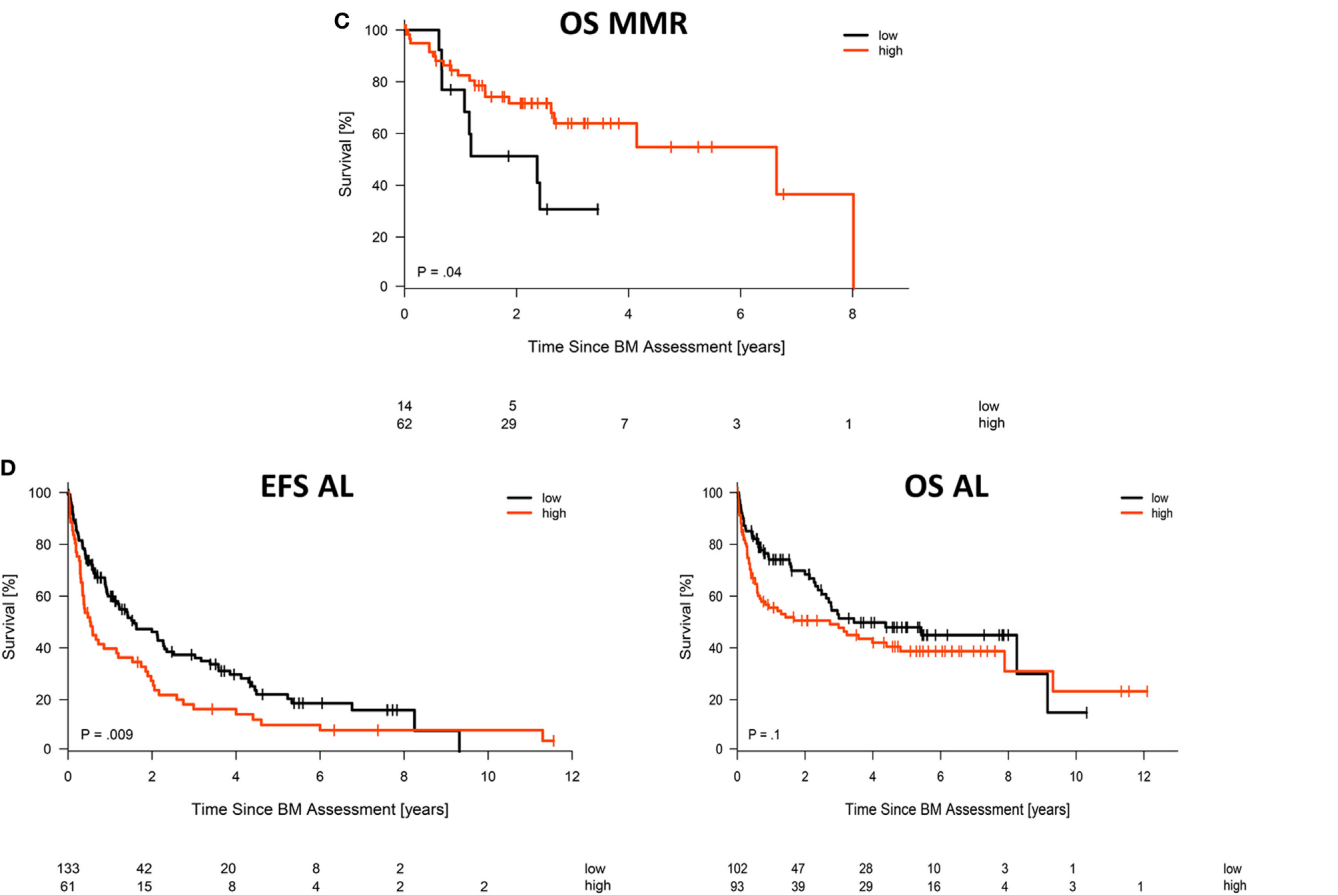
Association with survival. Impact of low vs. high CD38 expression according to gene expression profiling on **(A)** time to progression in asymptomatic myeloma patients, **(B1)** event-free and overall survival for our patient cohort treated with high-dose chemotherapy followed by autologous stem cell transplantation, including **(B2)** validation for patients treated within the total therapy 2 or 3 protocol, respectively, **(C)** overall survival of patients with relapsed/refractory myeloma, as well as **(D)** hematologic event-free and overall survival for patients with AL-amyloidosis. Patients with “high” and “low” CD38 expression in microarray data were delineated using maximally selected rank statistics with maxstat thresholds calculated for each entity: 13.34 (AMM), 11.64 (MM), 11.53 (MMR), and 13.15/12.95 (AL EFS/OS), respectively.

Asymptomatic patients with high CD38-expression (CD38^high^, delineated by logrank-based threshold) according to GEP, the largest cohort analyzed, had a significantly longer time to progression (*p* = 0.04; Figure 3A) and symptomatic patients had better event-free as well as overall survival (*p* = 0.03 and *p* = 0.02; Figure 3B1). For symptomatic patients, this was validated in the independent TT2/TT3 cohort from the University of Arkansas (*p* = 0.04 and *p* = 0.02; Figure 3B2). Better overall survival in case of CD38^high^ expression was likewise found for patients with relapsed/refractory myeloma (*p* = 0.04; Figure 3C). By contrast, AL-amyloidosis patients with CD38^high^ expressing malignant plasma cells did worse regarding hematologic event-free survival (*p* = 0.009) and a trend for overall survival (*p* = 0.1; Figure 3D). The relative hazard ratio of CD38-expression for symptomatic myeloma patients [including all cytogenetic entities, HR OS = 0.78 (0.6–1), *p* = 0.03] is comparable to those by adverse chromosomal aberrations, e.g., for del17p13 [HR OS = 1.53 (1.2–2), *p* = 0.002], t(4;14) [HR OS = 1.75 (1.4–2.3), *p* < 0.001], or 1q21 [HR OS = 1.65 (1.4–2) for >2 vs. 2 copies, *p* < 0.001, and HR OS = 2.23 (1.5–3.3) for >3 vs. 2 copies, *p* < 0.001], and altered gene expression, i.e., the UAMS70-gene score [HR OS = 1.94 (1.6–2.4), *p* < 0.001] or proliferation [HR OS = 1.4 (1.1–1.7) for medium vs. low risk, *p* = 0.001, and HR OS = 2.42 (1.8–3.2) for high vs. low risk, *p* < 0.001] (Table S2 in Supplementary Material). In multivariate analysis regarding event-free survival in multiple myeloma, CD38-expression (GEP) and the chromosomal aberrations t(4;14), del17p13, 1q21 (>2 and >3 copies) are independently prognostic, whereas t(11;14) and hyperdiploidy are not (Table S3 in Supplementary Material).

Chromosomal alterations showed different patterns of association with CD38-expression (according to GEP, the largest cohort analyzed; Tables 2 and 3; Figure S1 in Supplementary Material) in symptomatic myeloma. Whereas no difference was found for gain of 1q21 (2 vs. >2 vs. >3 copies), lower expression levels were found for del17p13 (*p* = 0.008) in contrast to higher CD38-expression in case of the presence of t(4;14) (*p* < 0.001). In the latter case, significant differences were found likewise for MGUS (*p* = 0.01) and asymptomatic myeloma (*p* = 0.002). Significantly lower CD38-expression was found for hyperdiploidy in symptomatic patients (*p* < 0.001) and for t(11;14) in asymptomatic myeloma (*p* = 0.006). Results were validated by RNA-seq for t(4;14) and hyperdiploidy (Tables 2 and 3; Figure S2A in Supplementary Material), the latter also in flow cytometric assessment (all patients; Tables 2 and 3; Figure S2B in Supplementary Material). Our iFISH- and ISS-based GMMG high-risk sore (19) did not show an association with CD38-expression.

**Table 2:** Expression levels of CD38 for different chromosomal aberrations in patients presenting with (yes) vs. without (no) the corresponding aberration.

		GEP					RNA-Seq					Flow cytometry				
Entity	Aberration	N yes	N no	Median exprs yes	Median exprs no	Difference	N yes	N no	Median exprs yes	Median exprs no	Difference	N yes	N no	Median exprs yes	Median exprs no	Difference
ALL	t(4;14)	154	1,197	13.12	12.57	0.553	58	529	9.00	8.49	0.509	59	699	5.97	5.91	0.055
ALL	del17p13	125	1,226	12.45	12.66	0.213	48	539	8.71	8.54	0.175	54	740	5.73	5.93	0.204
ALL	gain 1q21	503	833	12.63	12.63	0.008	225	360	8.45	8.58	0.126	267	527	5.72	6.00	0.280
ALL	t(11;14)	374	991	12.63	12.64	0.001	162	429	8.63	8.49	0.142	259	536	6.10	5.86	0.234
ALL	Hyperdiploidy	544	702	12.48	12.75	0.272	236	317	8.36	8.65	0.291	303	466	5.77	5.99	0.223
AL	t(4;14)	5	188	13.32	12.88	0.436	4	120	8.90	8.61	0.292	5	173	6.02	6.00	0.027
AL	del17p13	7	189	12.63	12.89	0.255	4	120	8.74	8.61	0.132	7	193	5.51	6.00	0.491
AL	gain 1q21	65	130	12.89	12.88	0.009	45	78	8.58	8.63	0.053	51	148	5.72	6.11	0.382
AL	t(11;14)	118	78	12.84	12.96	0.114	73	51	8.71	8.58	0.127	131	69	6.12	5.90	0.220
AL	Hyperdiploidy	38	157	12.93	12.88	0.047	23	101	8.37	8.63	0.262	36	163	5.70	6.04	0.346
MGUS	t(4;14)	4	53	13.60	12.89	0.707	4	43	9.39	8.53	0.862	7	83	6.65	6.21	0.442
MGUS	del17p13	0	60	–	12.99	–	0	51	–	8.68	–	1	96	6.68	6.23	0.452
MGUS	gain 1q21	18	42	13.06	12.97	0.092	17	34	8.45	8.72	0.276	18	79	6.04	6.27	0.237
MGUS	t(11;14)	9	51	13.00	12.94	0.061	8	42	8.72	8.61	0.108	16	82	6.50	6.18	0.312
MGUS	Hyperdiploidy	18	40	12.84	13.05	0.205	15	34	8.41	8.77	0.360	27	63	6.15	6.22	0.070
AMM	t(4;14)	31	223	13.05	12.60	0.449	6	23	8.89	8.23	0.655	17	176	5.72	5.80	0.074
AMM	del17p13	12	242	12.76	12.63	0.132	2	27	9.28	8.27	1,011	8	192	5.62	5.83	0.215
AMM	gain 1q21	83	166	12.63	12.65	0.016	11	16	8.06	8.55	0.484	63	136	5.64	5.90	0.259
AMM	t(11;14)	59	198	12.39	12.68	0.296	4	25	8.89	8.27	0.618	43	155	5.99	5.77	0.224
AMM	Hyperdiploidy	103	140	12.61	12.73	0.120	17	10	8.23	8.79	0.564	94	99	5.69	5.91	0.219
MM	t(4;14)	103	655	13.21	12.42	0.786	44	343	9.00	8.39	0.610	21	177	5.94	5.86	0.079
MM	del17p13	88	663	12.30	12.51	0.206	42	341	8.59	8.47	0.127	18	179	5.82	5.87	0.053
MM	gain 1q21	287	455	12.60	12.43	0.171	152	232	8.39	8.51	0.113	81	118	5.76	5.97	0.207
MM	t(11;14)	163	599	12.40	12.51	0.109	77	311	8.57	8.45	0.118	43	156	6.12	5.83	0.294
MM	Hyperdiploidy	346	317	12.43	12.66	0.228	181	172	8.35	8.59	0.240	101	91	5.82	5.95	0.130
MMR	t(4;14)	11	78	12.62	12.32	0.304	–	–	–	–	–	9	90	6.03	5.68	0.351
MMR	del17p13	18	72	12.40	12.38	0.024	–	–	–	–	–	20	80	5.75	5.68	0.070
MMR	gain 1q21	50	40	12.49	12.29	0.204	–	–	–	–	–	54	46	5.66	5.73	0.072
MMR	t(11;14)	25	65	12.44	12.35	0.091	–	–	–	–	–	26	74	5.69	5.68	0.010
MMR	Hyperdiploidy	39	48	12.26	12.49	0.232	–	–	–	–	–	45	50	5.63	5.72	0.083

*Log data of the median expression in GEP, RNA-seq, and flow cytometry are depicted for all patients together (ALL) as well as patients with AL-amyloidosis (AL), MGUS, asymptomatic (AMM), symptomatic (MM), and relapsed/refractory myeloma (MMR) separately*.

*GEP, gene expression profiling; MGUS, monoclonal gammopathy of unknown significance*.

**Table 3:** Differences in CD38 expression.

		ALL			AL			MGUS			AMM			MM			MMR		
		GEP	RNA-seq	Flow	GEP	RNA-seq	Flow	GEP	RNA-seq	Flow	GEP	RNA-seq	Flow	GEP	RNA-seq	Flow	GEP	RNA-seq	Flow
iFISH	t(4;14)																	NA	
	del17p13																	NA	
	gain 1q21																	NA	
	t(11;14)																	NA	
	Hyperdiploidy																	NA	

GEP-scores	GPI		NA			NA			NA			NA			NA			NA	
	UAMS70		NA			NA			NA			NA			NA			NA	
	Rs		NA			NA			NA			NA			NA			NA	
	EMC92		NA			NA	NA		NA	NA		NA			NA			NA	

Tumor mass	ISS																	NA	
	rISS																	NA	
	GMMG high-risk score																	NA	
	PCI							NA	NA	NA								NA	
	FLCR							NA	NA	NA								NA	
	MRI	NA	NA	NA	NA	NA	NA	NA	NA	NA				NA	NA	NA	NA	NA	NA
	Kyle model				NA	NA	NA	NA	NA	NA								NA	
	# of affected organs	NA	NA	NA				NA	NA	NA	NA	NA	NA	NA	NA	NA	NA	NA	NA
	Heart involvement	NA	NA	NA				NA	NA	NA	NA	NA	NA	NA	NA	NA	NA	NA	NA
	NT-proBNP	NA	NA	NA				NA	NA	NA	NA	NA	NA	NA	NA	NA	NA	NA	NA
	Mayo 2004 score	NA	NA	NA				NA	NA	NA	NA	NA	NA	NA	NA	NA	NA	NA	NA

*Differences in CD38 expression with regards to the presence of chromosomal aberrations, gene expression-based risk scores, and clinical parameters as surrogates of tumor mass according to GEP, RNA-sequencing, and flow cytometry for all patients together (ALL) as well as patients with AL-amyloidosis (AL), MGUS, asymptomatic (AMM), symptomatic (MM), and relapsed myeloma (MMR) separately. A significant downregulation of CD38 is depicted in red color, a significant upregulation in green. Gray color indicates non-significant differences. NA, not assessed. Dark red and dark green depict significant *p* values after Benjamini–Hochberg correction*.

*MGUS, monoclonal gammopathy of unknown significance; GEP, gene expression profiling; iFISH, interphase fluorescence *in situ* hybridization; (r)ISS, (revised) International Staging System; PCI, plasma cell infiltration; FLCR, free light chain ratio; MRI, magnetic resonance imaging (see Supplementary Material for details)*.

From investigated gene expression-based factors including proliferation, only the UAMS70-gene-score showed an association with CD38-expression in symptomatic myeloma patients (*p* < 0.001); surprisingly, high risk correlated with significantly *higher* CD38-expression (Table 3; Figure S3 in Supplementary Material). This association could be validated in the independent TT2/TT3 cohort.

Correlation of CD38-expression with prognostic clinical parameters was neither observed in symptomatic nor asymptomatic myeloma patients in more than one assessment of CD38-expression (Table 3; Figures S4–S6 in Supplementary Material).

In patients with AL-amyloidosis, CD38-expression is neither associated with the number of affected organs, involvement of the heart as the most critical organ, nor the surrogate marker NT-proBNP (<332 vs. ≥332 ng/l) or the Mayo2004-score (Table 3; Figures S4–S6 in Supplementary Material). Patients in the CD38^high^ expressing cohort in terms of overall survival (maxstat cutoff) showed slightly but significantly higher NT-proBNP-levels (*p* = 0.04).

### Potential Mechanisms of Primary Resistance

**Figure 4:**
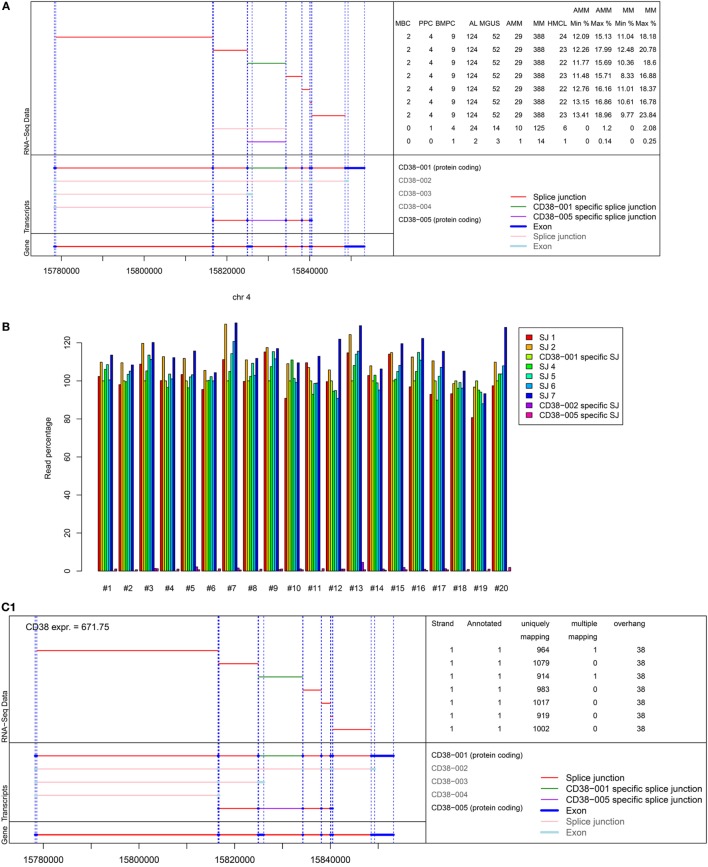

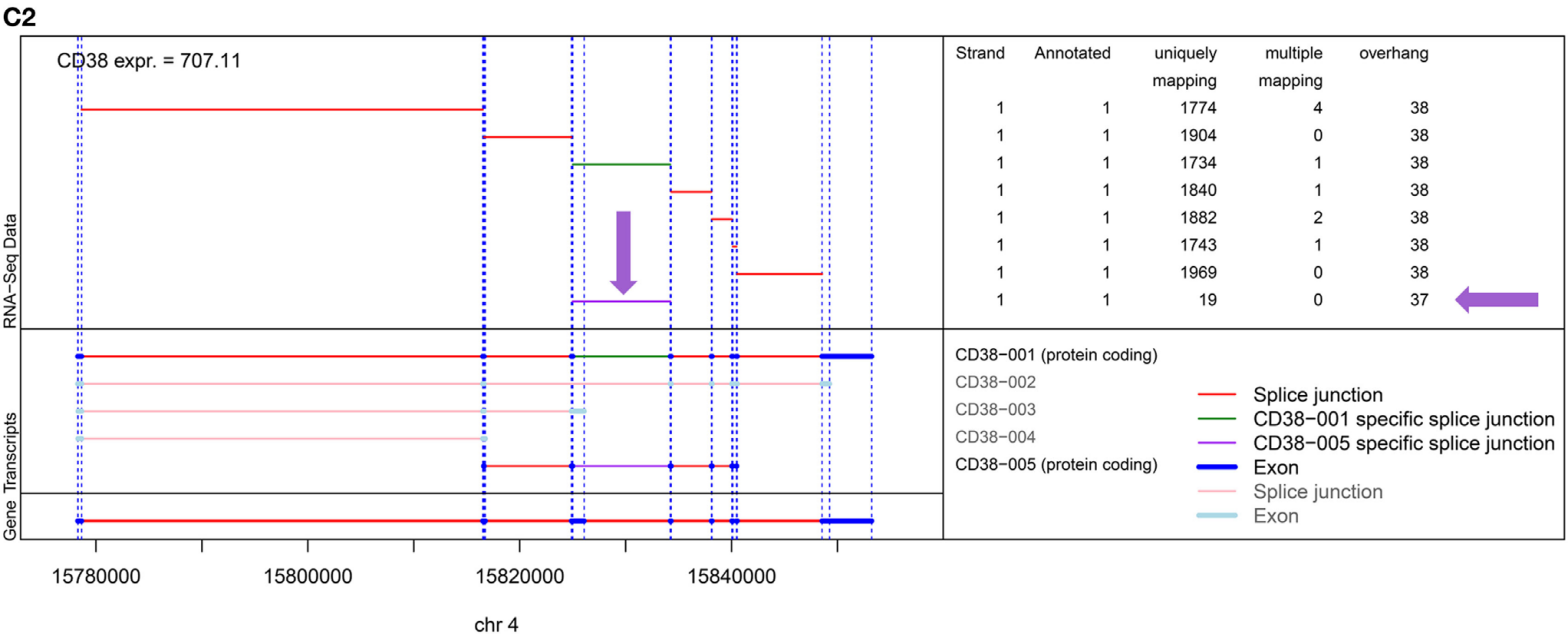
Analysis of alternative splicing of CD38. Five splice variants for CD38 are annotated in GRCh38, two of which being protein encoding, i.e., CD38-001 and CD38-005, focused on here. Of the three remaining transcripts, CD38-003 and CD38-004 are both non-protein coding due to retained intron sequences, and CD38-002 shows nonsense-mediated decay. **(A)** Shown is the structure of the CD38 locus (bottom left) followed by the different transcripts (*n* = 5) with splice junctions (*n* = 9) depicted in red and exons (*n* = 17) depicted in dark blue. Eight exons and seven splice junctions belong to CD38-001 and one junction exists only in CD38-001, depicted in green. The splice junction specific for CD38-005 is depicted in lilac. This is likewise explained in the bottom right part of the figure. The left part of the top panel shows the individual splice junctions expressed in RNA-seq analysis. The right part of the top panel gives the number of samples expressing the respective splice junction. The splice junction specific for CD38-001 is expressed in all 593 plasma cell samples while the one for CD38-005 is expressed only in 2/124 AL-patients (1.6%), 3/52 MGUS (5.8%), 1/29 AMM (3.4%), and 14/388 MM patient samples (3.6%), respectively. The last four columns give the minimum (min) and maximum (max) percentage of raw reads for the specific junctions compared to all reads spanning a CD38 splice junction in asymptomatic and symptomatic myeloma patients. **(B)** Given is the relative abundance of reads per splice junction in comparison to CD38-001 specific splice junction for the 20 patients in whom CD38-005 (lilac) supported by at least 10 reads was found. The maximum observed frequency is 1.8%. **(C)** Exemplary data for **(C1)** a patient showing expression of CD38-001 only. All seven splice junctions and eight exons are present. Reads for individual splice junctions are depicted to the right. **(C2)** Exemplary patient showing additional alternative splicing in terms of expression of CD38-005 but with low frequency (lilac arrows). In the depicted patient, the relative abundance of this specific junction compared to that of CD38-001 is 1.1%. See also Table S3 in Supplementary Material. RSEM transcript analysis for the same patients confirming the data is shown in Figure S7 in Supplementary Material.

As we could exclude lack of CD38-expression as potential mechanism of upfront resistance to single-agent anti-CD38 treatment (Figure 1; Table 1), we investigated alternative CD38-splicing potentially leading to the removal of the antibody binding site. We assessed the five CD38 transcripts annotated in GRCh38 in terms of the presence and abundance of (specific) splice junctions. We focused on the two protein coding transcripts CD38-001 and CD38-005, both of which can be identified by a transcript specific splice junction. The full-length eight-exon-transcript CD38-001 was expressed in all malignant plasma cell samples (Figure 4A). It was by far the most abundant protein coding transcript with >97% of associated reads in CD38-specific splice junctions in all individual malignant and normal plasma cell samples (Table 4). CD38-005 transcript specific splice junction with a read count above 10 was detected in 20/593 (3.4%) patient samples with a maximum frequency of 1.8% compared to that of CD38-001 (Figure 4B). Exemplary data are shown in Figure 4C.

**Table 4:** Alternative splicing of CD38 and relative abundance of protein coding transcripts.

Population	*n*	CD38-001	% CD38-001	CD38-001 only	% CD38-001 only	Number of annotated reads other than CD38-001		
						>1%	>5%	>10%
MBC	4	1	25.0	1	25.0	0	0	0
PPC	4	4	100	2	50.0	0	0	0
BMPC	10	10	100	3	33.3	0	0	0
AL	124	124	100	96	77.4	0	0	0
MGUS	52	52	100	36	69.2	3	0	0
AMM	29	29	100	17	58.6	0	0	0
MM	388	388	100	247	63.7	1	0	0
HMCL	26	22	84.6	15	57.7	0	0	0

*Shown is the percentage of samples with detection of the full-length eight-exon transcript CD38.001 (protein coding) in comparison to the percentage of samples with detection of any other CD38 transcript (including non-protein coding) above the levels of 1, 5, and 10%, respectively. The highest non-CD38-001 frequency found in any of the samples is 2.1%, the highest frequency of the alternate protein-coding transcript CD38-005 is 1.8%*.

*MBC, memory B-cells; PPC, polyclonal plasmablastic cells; BMPC, (normal) bone marrow plasma cells; AL, AL-amyloidosis; MGUS, monoclonal gammopathy of unknown significance; (A)MM, (asymptomatic) multiple myeloma; MMR, relapsed/refractory myeloma; HMCL, human myeloma cell line*.

Results are confirmed by RSEM transcript analysis. Here, a high number of mapping reads is present for transcript 1 compared with few for transcript 5. The analysis shows a gap in matching reads for transcript 5 between position 161 and 162, i.e., exactly the position of the transcript 5 specific splice junction; reads overlapping this splice junction are missing (see Figure S7 in Supplementary Material for exemplary data).

## Discussion

Monoclonal antibodies against the cell surface antigen CD38, e.g., isatuximab, daratumumab, or Mor202, have entered the therapeutic armamentarium in multiple myeloma. Of these, daratumumab is approved for the treatment of relapsed/refractory myeloma in Europe and the US. Daratumumab is likewise approved in the US for the upfront-treatment of non-transplant eligible patients in combination with melphalan and prednisone based on recent data from the ALCYONE trial (47). Trials with daratumumab and other anti-CD38 antibodies are ongoing for upfront treatment of symptomatic (e.g., NCT03012880 and NCT02541383) and asymptomatic/smoldering myeloma (e.g., NCT02316106) as well as AL-amyloidosis (6). In the latter disease, treatment requires a quick reduction of the malignant cell clone to stop amyloid production and organ deposition, but amyloid-associated morbidities frequently limit intensity of treatment (48, 49).

In our study, we fist addressed expression of CD38 in a large cohort of patients with different stages of plasma cell diseases. We find that CD38 is expressed on malignant plasma cells of all entities, including AL-amyloidosis, with two-log fold variation of expression within the respective entities. From a methodological point of view, although not being contradictory, results obtained with different methods do not show complete overlap in terms of significant differences for all cohorts investigated. We see two main explanations. First, methods assessing abundance of mRNAs (GEP, RNA-sequencing) vs. those addressing protein surface expression (flow cytometry). Methodological differences are likewise present for the first two methods; whereas for GEP (microarrays) the number of binding sites and thus corresponding fluorescence intensity is limited and saturation effects come into place with high expression, this is not the case for RNA-seq where the actual number of transcripts (reads) is measured (with normalization to allow comparison between different samples). Observed significant differences are secondly impacted by the number of samples assessed by the respective method.

We next investigated the association of CD38-expression with molecular entities and patient survival. An interesting question to this end is, at what stage the downregulation compared to normal plasma cells appears. A significant downregulation is found between MGUS and asymptomatic myeloma stage, but not between subsequent stages thereafter. The latter can only be seen for comparison of longitudinal cohorts of asymptomatic/symptomatic or symptomatic/relapsed myeloma with small differences. Expression wise, there is thus no obvious preferential timing of anti-CD38 treatment. A further analysis was triggered when our GMMG-study group planned the CONCEPT trial (EudraCT 2016-000432-17) for upfront treatment of previously untreated high-risk myeloma patients [defined by the presence of t(4;14), del17p13, or >3 copies of 1q21 and ISS-stage II or III (19)] with addition of the anti-CD38-antibody isatuximab to intensified treatment. As no data were available, we considered it prudent to investigate whether in a subfraction of high-risk patients CD38-expression might be absent, potentially reducing anti-CD38 treatment efficacy. This is, however, not the case.

It is interesting to briefly discuss the potentially confusing associations of CD38-expression with survival and molecular entities. First, lower CD38-expression is associated independently with faster progression of asymptomatic to symptomatic and symptomatic to relapsed myeloma. At the same time, CD38-expression (according to GEP, the largest cohort analyzed) in symptomatic myeloma showed different patterns of association with adverse prognostic aberrations. Whereas no difference was found for gain of 1q21, lower levels of expression were found for del17p13, in contrast to higher CD38-expression in case of presence of t(4;14) independently for symptomatic myeloma, asymptomatic myeloma, and MGUS. Based on our multivariate analysis, CD38-expression and the chromosomal aberrations del17p13, t(4;14), and gain of 1q21 are independent. Adverse chromosomal aberrations can thus be associated with higher or lower CD38-expression, i.e., the intrinsic prognostic impact of these aberrations is either ameliorated by higher CD38-expression (good prognosis) or aggravated, as for del17p13 and lower CD38-expression (adverse prognosis). Both parameters can thus lead in the same or different prognostic direction. The relative hazard ratio of CD38-expression (symptomatic myeloma, including all cytogenetic entities) was comparable to those by adverse chromosomal aberrations and altered gene expression. In other words, the beneficial effect of high CD38-expression as potential surrogate for less dedifferentiated plasma cells is independent and only partially overcomes (if, in different prognostic direction) the adverse impact of chromosomal aberrations and altered gene expression. Analogously, significantly lower CD38-expression was found for t(11;14) in asymptomatic myeloma, associated in this entity with slower progression (data not shown), and hyperdiploidy in symptomatic patients; here with different prognostic directions: while hyperdiploidy is associated with good/standard risk (50, 51), low CD38-expression is associated with adverse prognosis.

In AL-amyloidosis, we found the opposite association: higher CD38-expression is associated with adverse survival. What are potential explanations? AL-amyloidosis is a different entity. First, treatment regimen can show different outcome in AL-compared to multiple myeloma patients, e.g., t(11;14) carrying AL-amyloidosis patients lack benefit from bortezomib-based treatment (49). Second, prognosis in AL-amyloidosis is mainly driven by the pattern (organotropism) and amount of AL-amyloid deposition, which outweigh malignant plasma cell features (42, 48). The amount of AL-amyloid deposition is driven by the number of malignant plasma cells, their individual light chain production, and the amyloidogeneic potential of an individual patient’s light chains. AL-patients with higher CD38 expression show slightly but significantly higher serum NT-proBNP-levels and thus cardiac involvement. Immunoglobulin or light chain production by malignant plasma cells in turn is significantly lower compared to normal plasma cells and can degrade with evolvement or plasmablastic disease (52). At the same time, we find a downregulation of CD38-expression in malignant compared to normal plasma cells that increases with more advanced stages, in relapse, and HMCLs, i.e., the degree of derangement from normal plasma cells. Patients with advanced disease show a higher frequency of molecular high-risk features, adversely associated with prognosis (13, 53). Taken together, we hypothesize that the higher level of AL-amyloid production per cell in less “abnormal” (differentiated) plasma cells (with concomitantly higher CD38-expression) thus outweighs the potentially beneficial association with a less deranged molecular phenotype.

What do we know about the mechanism of upfront resistance, i.e., why do two-thirds of relapsed/refractory myeloma patients not reach at least a partial remission (1, 4, 5)? From proposed mechanisms, we can first exclude complete absence of CD38-expression in agreement with previous reports of smaller cohorts and mainly relapsed patients (7–9, 11). Second, we show for the first time that expression of a truncated CD38-molecule with removed antibody binding-sequence by alternative splicing does not occur; of the two protein coding transcripts CD38-001 (eight-exon full length) and CD38-005 (truncated), non-CD38-001 transcripts are very rare (see Figure 4). Given that, e.g., daratumumab binds to amino acid (aa) 267–280 at the C-terminal region of human CD38 in combination with aa 233–246 (assessment report EMA/278085/2016), and one of these (aa 233–246) is encoded by both CD38-001 and CD38-005, whereas the other (aa 267–280) is found in CD38-001 only, daratumumab would not bind to the protein transcript encoded by CD38-005. Third, Nijhof et al. have shown that inhibition of the immune response due to the presence of inhibitory cell surface proteins on malignant plasma cells, e.g., CD46, CD55, or CD59, are neither associated with daratumumab-mediated complement-dependent cytotoxicity, nor the clinical response to the antibody (7). These three mechanisms can thus not be the culprit. The likely explanation seems more subtle: *relative* expression height of CD38 is reported as being important for anti-CD38 activity: *in vitro*, daratumumab-mediated antibody-dependent cellular and complement-dependent cytotoxicity is associated with expression of CD38 on target cells (54). *In vivo*, relapsed myeloma patients reaching a partial remission or better vs. responding less to daratumumab-treatment show higher CD38-expression on myeloma cells (7), but no “threshold” for anti-CD38 activity was found. Indeed, we find a two-log variation between CD38-expression in malignant plasma cell dyscrasias, with median expression in none of the entities reaching the level of normal plasma cells. Taken together, these findings suggest an association but no absolute correlation (threshold) of CD38-expression as background of reduced activity.

Relative association of CD38-expression and anti-CD38 response have prompted efforts investigating pharmacological upregulation of CD38 during anti-CD38 exposure, e.g., by using all-trans retinoic acid (54) or panobinostat (55). Median CD38-expression levels significantly below normal plasma cells in all entities in our study give further evidence for potential pharmacological upregulation of CD38 concomitantly with anti-CD38 treatment.

In conclusion, CD38 is expressed in all plasma cell dyscrasias with two-log variation within the entities, though at lower level compared to normal plasma cells. High CD38-expression is associated with lower risk of progression from asymptomatic to symptomatic and symptomatic to relapsed myeloma and better overall survival in the latter two. By contrast, high CD38-expression is prognostically adverse in AL-amyloidosis. Upfront resistance against anti-CD38 treatment is neither mediated by (complete absence) of CD38-expression nor alternative splicing removing the antibody target sequence, but likely only influenced by a gradual difference in expression levels.

## Ethics Statement

This study was carried out in accordance with the recommendations of the ethics board of the Medical Faculty Heidelberg. The protocol was approved by the ethics committee (#229/2003, #S-152/2010). All subjects gave written informed consent in accordance with the Declaration of Helsinki.

## Author Contributions

Conception and design: DH and AS. Provision of study materials or patients: JH, DH, CK, TD, MH, M-SR, ADH, UH, and SS. Collection and assembly of data: AS, ME, SB, CK, TD, AJ, UH, SS, and DH. Data analysis and interpretation: DH, AS, JH, and SS. Manuscript writing: AS and DH. Final approval of manuscript: all authors.

## Conflict of Interest Statement

The authors declare research funding from Sanofi. Sanofi was not involved in the study design, interpretation of data, or writing of the manuscript.

## Footnotes

^1^https://www.r-project.org/.

^2^https://www.bioconductor.org/.

^3^http://cran.r-project.org/web/packages/maxstat/index.html.
