# Supplementary material for: CD38 as Immunotherapeutic Target in Light Chain Amyloidosis and Multiple Myeloma—Association With Molecular Entities, Risk, Survival, and Mechanisms of Upfront Resistance
Source: Front Immunol. 2018 Jul 20;9:1676. doi: 10.3389/fimmu.2018.01676 (PMC6062598; doi:10.3389/fimmu.2018.01676)
Supplement: Supplementary file 1 [file data_sheet_1.PDF]

SUPPLEMENTARY DATA

Supplement to: Anja Seckinger, Jens Hillengass, Martina Emde, Susanne Beck, Christoph Kimmich, Tobias Dittrich, Michael Hundemer, Anna Jauch, Ute Hegenbart, Marc-Steffen Raab, Anthony D. Ho, Stefan Schönland, and Dirk Hose.

CD38 as Immunotherapeutic Target in Light Chain Amyloidosis and Multiple Myeloma - Association with Molecular Entities, Risk, Survival, and Mechanisms of Upfront Resistance.

SUPPLEMENTARY FIGURES

Supplementary Figure S1. Expression of CD38 in malignant plasma cells in relation to chromosomal aberrations.

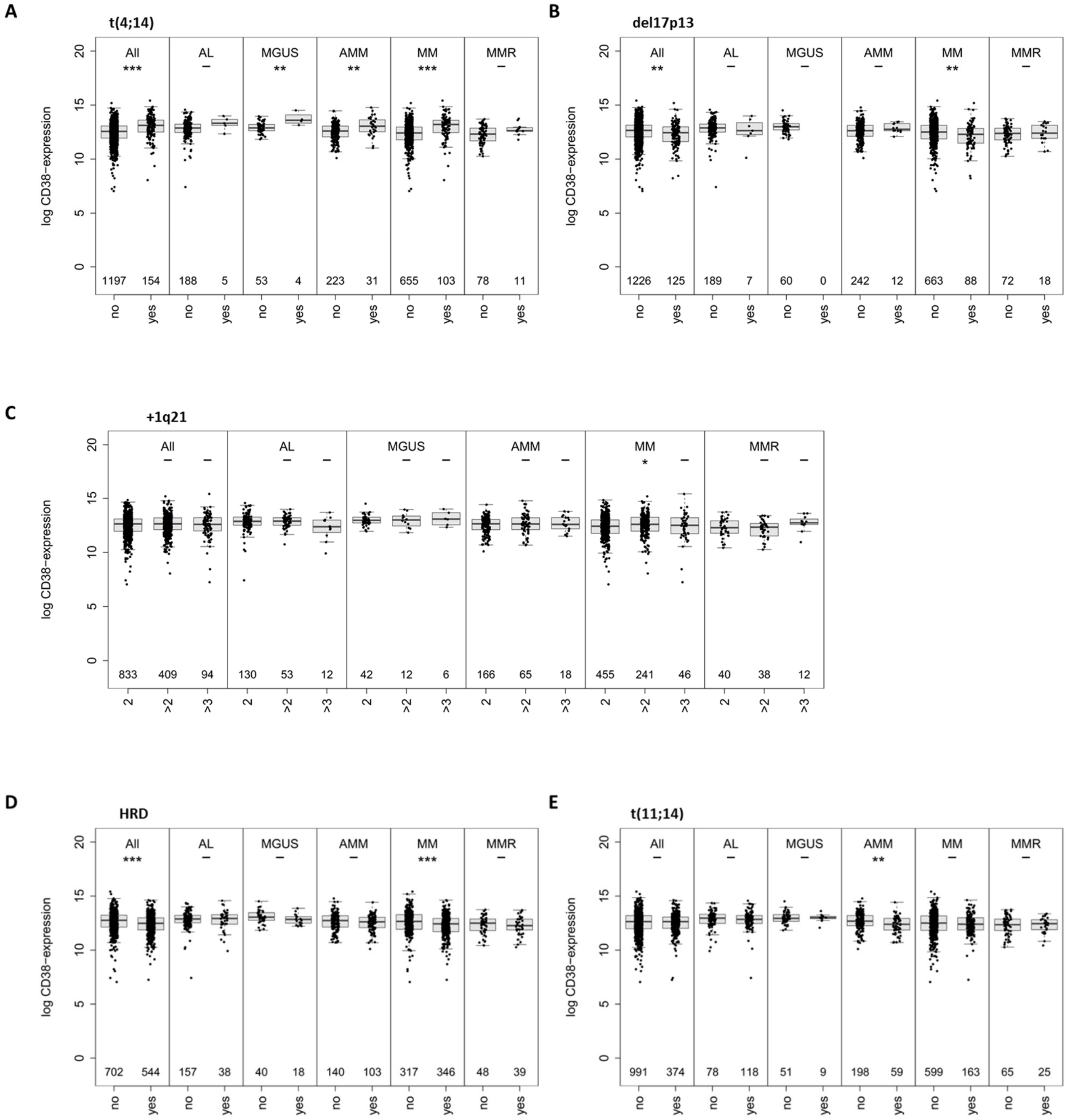

Association of CD38 expression as assessed by gene expression profiling using DNA-microarrays of malignant plasma cells from patients with AL-amyloidosis (AL), MGUS, AMM, MM, and MMR and chromosomal aberrations as assessed by interphase fluorescence in-situ hybridization regarding

presence chromosomal aberrations associated with adverse prognosis in symptomatic myeloma, i.e. (**A**) t(4;14), (**B**) del17p13, and (**C**) 1q21 gain. Chromosomal aberrations associated with etiology, i.e. (**D**) hyperdiploidy (HRD) and (**E**) t(11;14). Figures depict the presence (yes) vs. absence (no) of the respective aberration. In case of gain of 1q21, copy numbers are depicted, i.e. 2 (normal diploid stage) vs. >2 vs. >3 copies. The first panel depicts all patients, the subsequent panels AL, MGUS, AMM, MM, and MMR. Significant difference between the groups is depicted by one asterisk (*) for a level of $p < 0.05$, two asterisks (**) for a level of $p < 0.01$, and three (***) for $p < 0.001$ with corresponding patient numbers being depicted in the boxplots. For the same analysis performed using RNA-sequencing and flow cytometry, see Figure S2. Table 2 depicts height of CD38 expression in patient presenting with the respective aberration vs. those without and corresponding expression differences.

Supplementary Figure S2. Expression of CD38 in malignant plasma cells with regards to chromosomal aberrations.

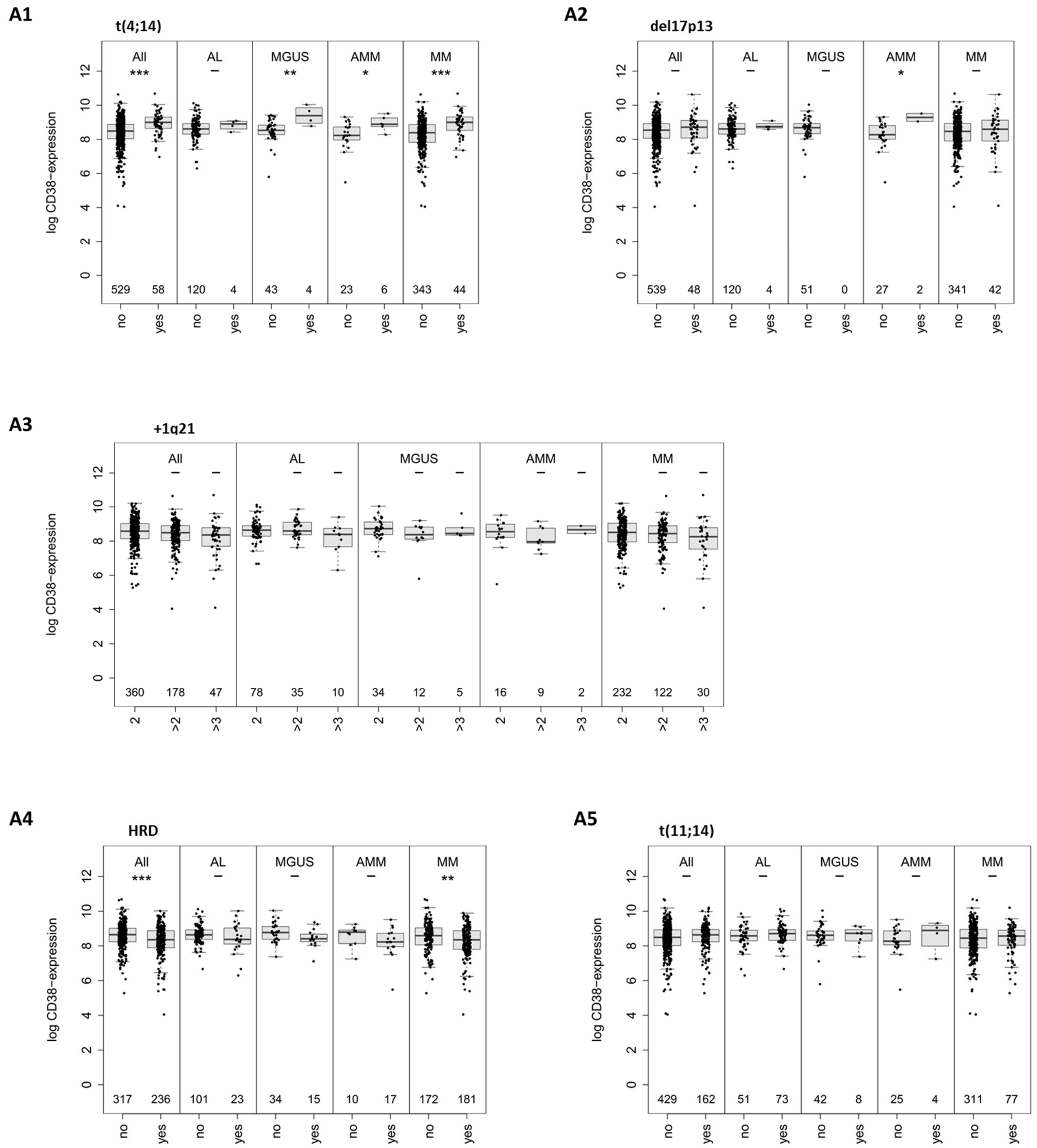

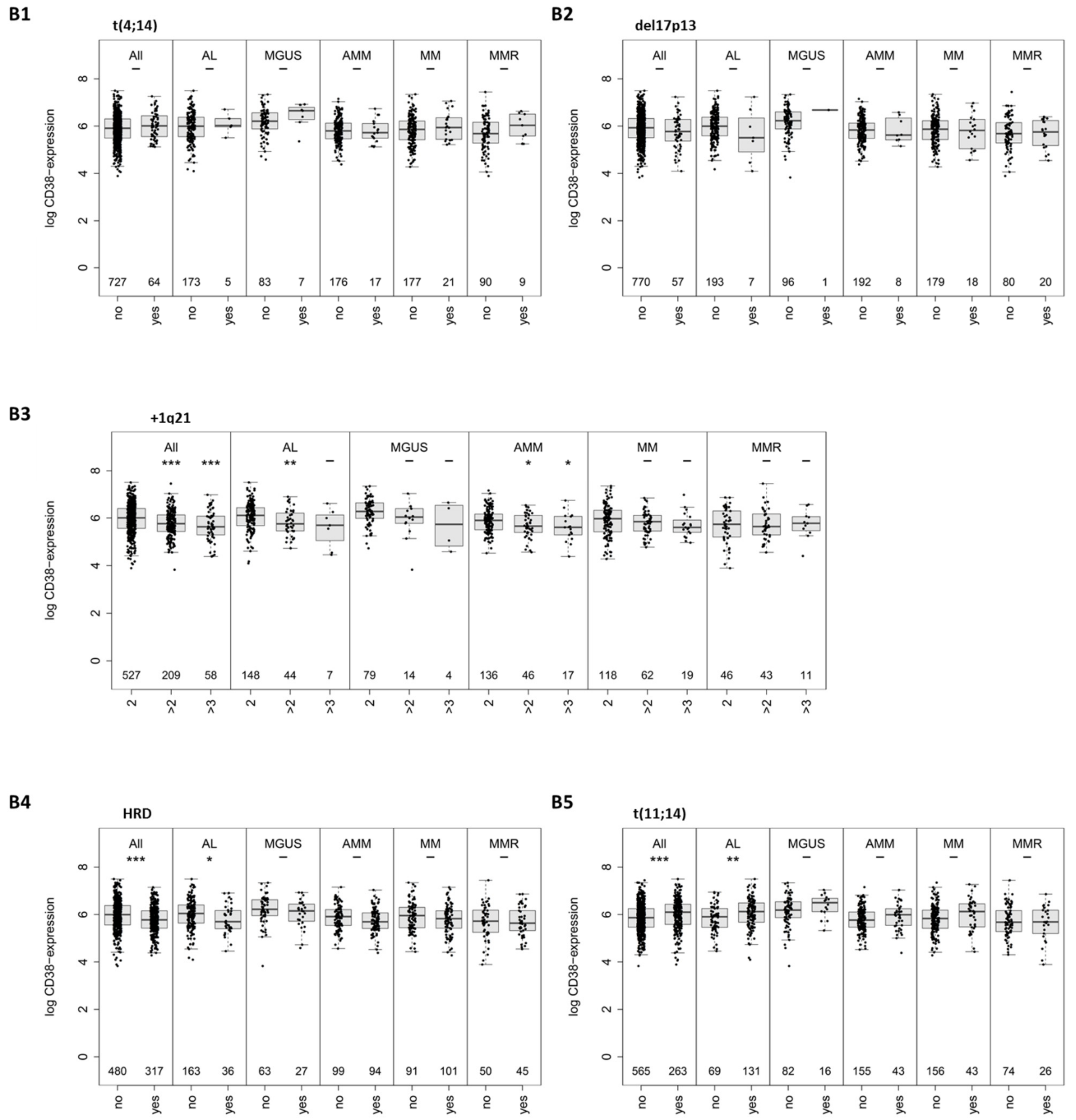

Association of CD38 expression by (**A**) RNA-seq and (**B**) flow cytometry of malignant plasma cells from patients with AL-amyloidosis (AL, n = 124/200), monoclonal gammopathy of unknown significance (MGUS, n = 52/100), asymptomatic myeloma (AMM, n = 29/200), as well as symptomatic myeloma (MM, n = 388/200) and chromosomal aberrations as assessed by interphase fluorescence in-situ hybridization regarding presence of high risk aberrations (A1), (B1) t(4;14), (A2), (B2) del17p13, and (A3), (B3) gain 1q21, as well as presence of (A4), (B4) hyperdiploidy (HRD), and

(A5), (B5) t(11;14). But for 1q21 (2 vs. >2 vs. >3 copies), figures depict the presence (yes) vs. absence (no) of the respective aberration. The first panel depicts all patients, the subsequent panels the respective entities. Significant difference is depicted by one asterisk (*) for a level of $p < 0.05$, two asterisks (**) for a level of $p < 0.01$, and three (***) for $p < 0.001$ with corresponding patient numbers being depicted in the boxplots.

Supplementary Figure S3. Expression of CD38 in malignant plasma cells with regards to gene expression-based risk-scores and classifications.

A

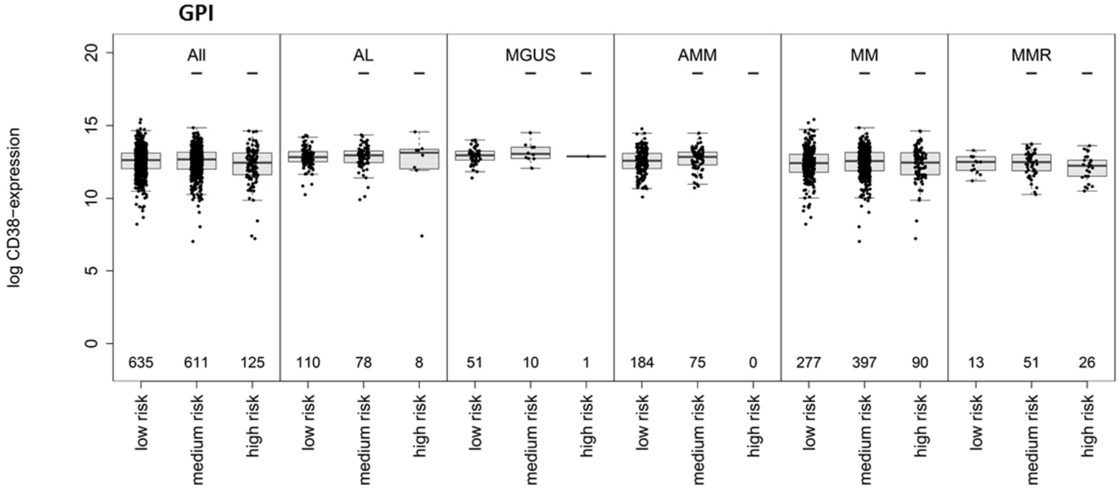

B

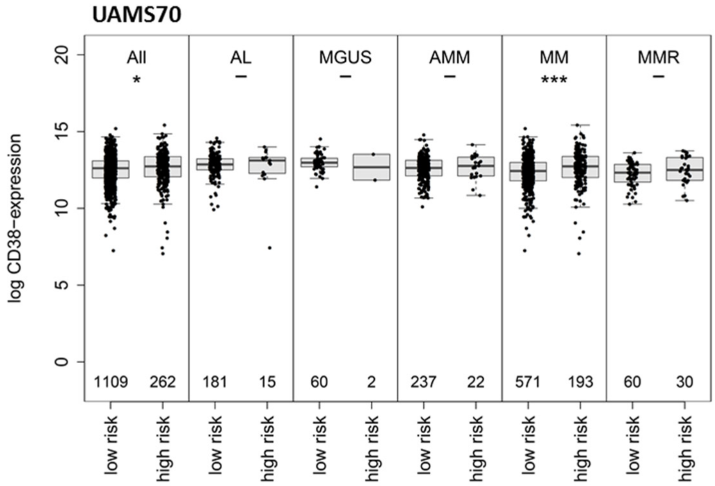

C

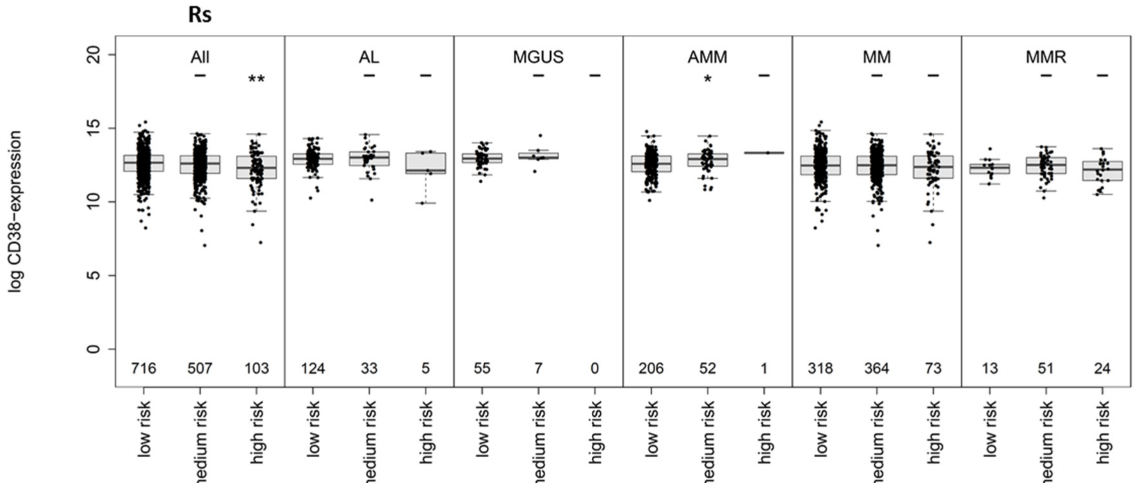

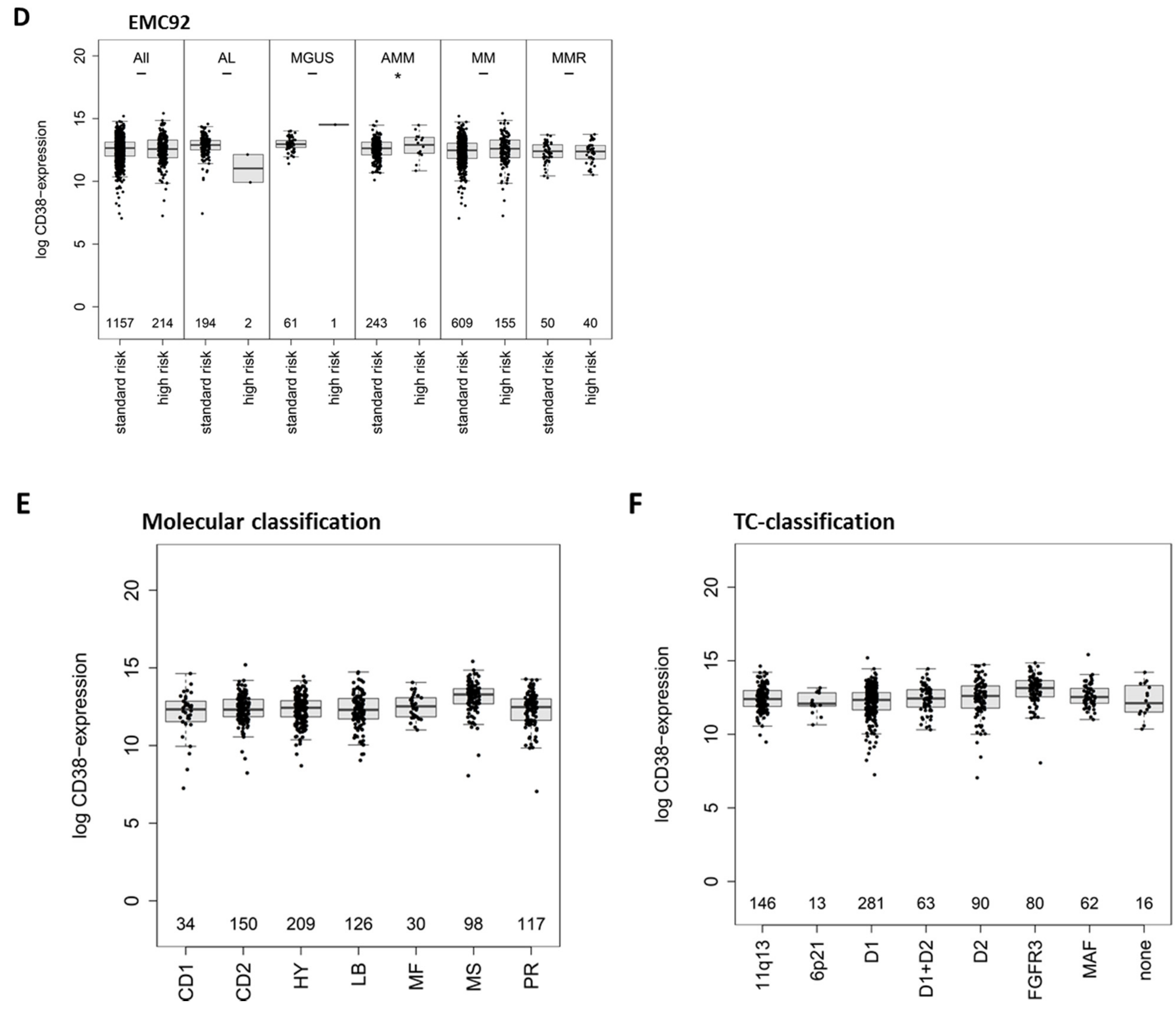

Depicted is the association of CD38 expression as assessed by global gene expression profiling of malignant plasma cells from patients with AL-amyloidosis (AL, n = 196), monoclonal gammopathy of unknown significance (MGUS, n = 62), asymptomatic myeloma (AMM, n = 259), symptomatic (MM, n = 764), as well as relapsed/refractory multiple myeloma (MMR, n = 90) and gene expression based risk-scores and classifications (for MM only), as well as proliferation. (**A**) Gene expression-based proliferation index (GPI; low vs. medium vs. high proliferation), (**B**) UAMS70-gene-score (low vs. high risk), (**C**) Rs-score (low vs. medium vs. high risk), (**D**) EMC92-gene-score (standard vs. high risk), (E) molecular classification, and (F) TC-classification. Significant difference between the groups

is depicted by one asterisk (*) for a level of $p < 0.05$, two asterisks (**) for a level of $p < 0.01$, and three (***) for $p < 0.001$ with corresponding patient numbers being depicted in the boxplots.

Supplementary Figure S4. Expression of CD38 based on gene expression profiling in malignant plasma cells with regards to clinical parameters.

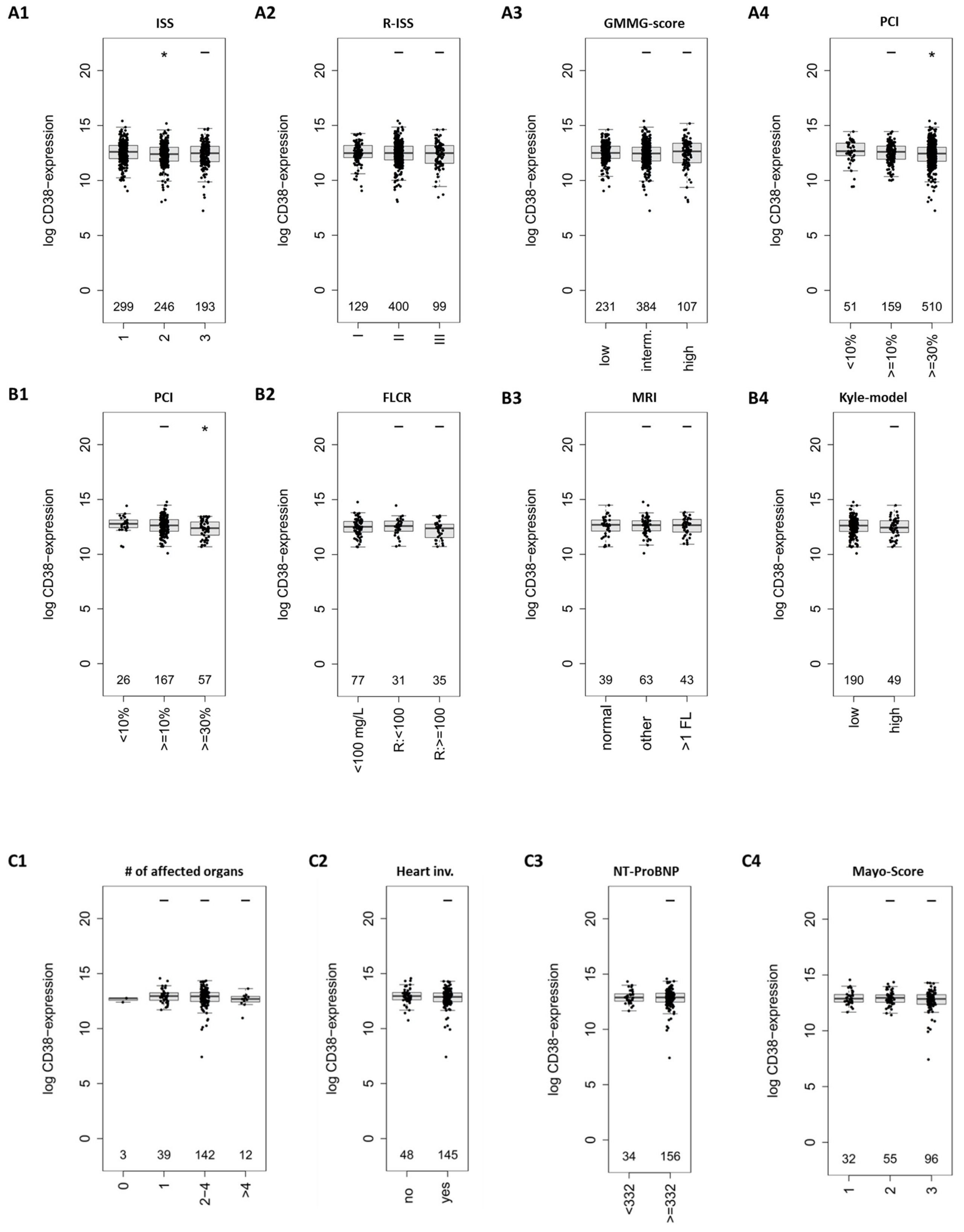

CD38-expression as assessed by gene expression profiling using DNA-microarrays and association with clinical risk factors and surrogates of tumor mass in (**A**) symptomatic myeloma. Shown are (A1) ISS-stage (I vs. II vs. III), (A2) revised ISS-stage (I vs. II vs. III), (A3) GMMG-score (low vs. intermediate vs. high risk), and (A4) plasma cell infiltration (PCI; <10% vs. ≥10% vs. ≥30%). (**B**) Asymptomatic myeloma. Shown are local and global surrogates of tumor mass, i.e. (B1) plasma cell infiltration (<10% vs. ≥10% vs. ≥30%), (B2) free light chain ratio, (B3) presence of alterations in whole body MRI (wb-MRI), i.e. presence of >1 focal lesion vs. other alterations vs. no alterations (according to (1)), and (B4) tumor mass according to Kyle *et al*. (low vs. high). (**C**) AL-amyloidosis. Shown are organ involvement with regards to (C1) number of affected organs (1 vs. 2-4 vs. >4), (C2) presence of heart involvement, (C3) NT-proBNP level (<332 ng/l vs. ≥332 ng/l), and (C4) the Mayo2004-score (1 vs. 2 vs. 3). Significant difference between the groups is depicted by one asterisk (*) for a level of $p < 0.05$ with corresponding patient numbers being depicted in the boxplots. For the same analysis performed using RNA-sequencing and flow cytometry, see Supplementary Figures S5 and S6.

Supplementary Figure S5. Expression of CD38 based on RNA-sequencing in malignant plasma cells with regards to clinical parameters.

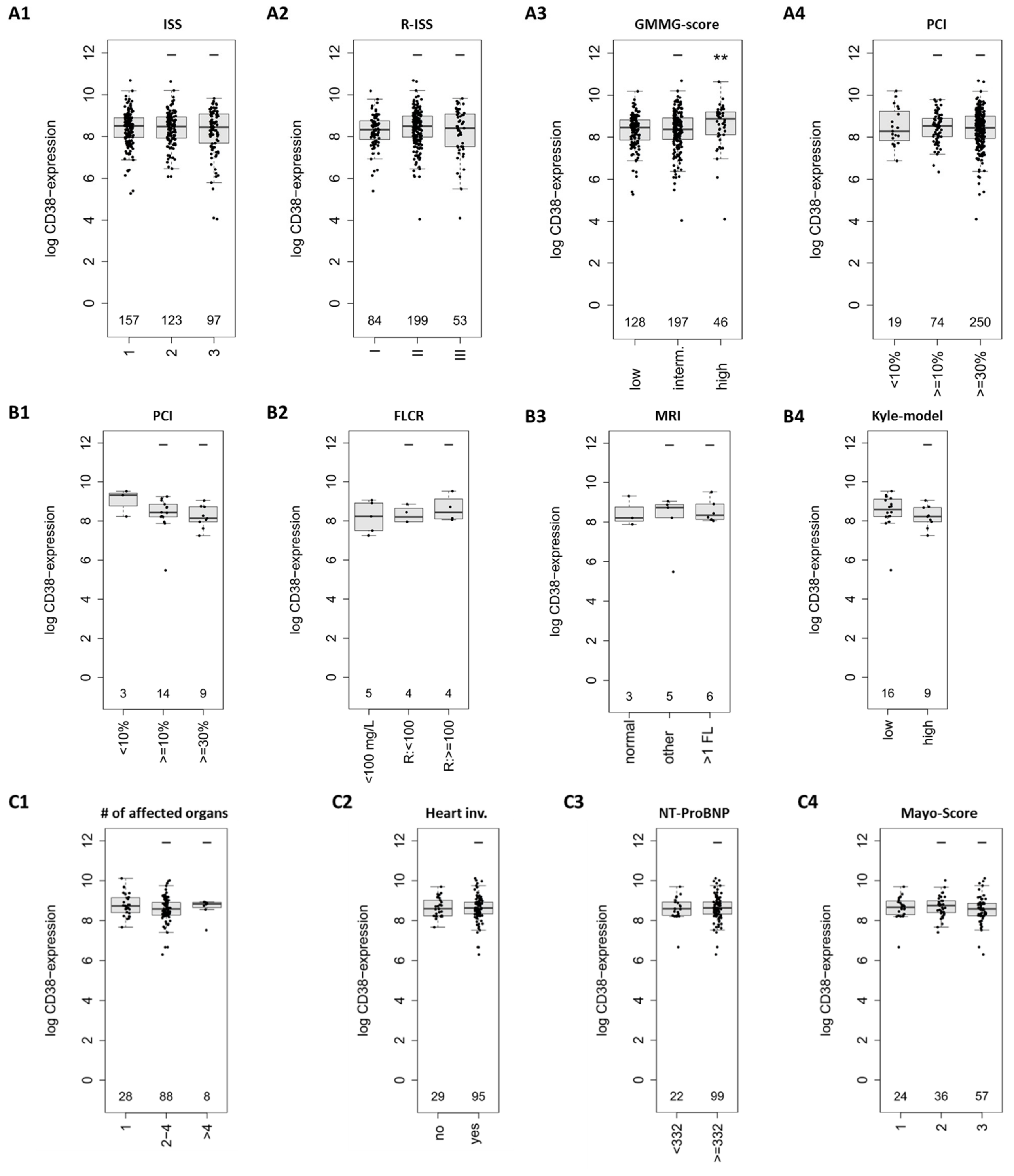

CD38-expression assessed by RNA-seq and association with clinical risk factors and surrogates of tumor mass in (**A**) symptomatic myeloma. Shown are (A1) ISS-stage (I vs. II vs. III), (A2) revised ISS-stage (I vs. II vs. III), (A3) GMMG-score (low vs. intermediate vs. high risk), and (A4) plasma cell

infiltration (PCI; <10% vs. ≥10% vs. ≥30%). (**B**) Asymptomatic myeloma. Shown are local and global surrogates of tumor mass, i.e. (B1) plasma cell infiltration (<10% vs. ≥10% vs. ≥30%), (B2) free light chain ratio, (B3) presence of alterations in whole body MRI (wb-MRI), i.e. presence of >1 focal lesion vs. other alterations vs. no alterations (according to (1)), and (B4) tumor mass according to Kyle *et al*. (low vs. high). (**C**) AL-amyloidosis. Shown are organ involvement with regards to (C1) number of affected organs (1 vs. 2-4 vs. >4), (C2) presence of heart involvement, (C3) NT-proBNP level (<332 ng/l vs. ≥332 ng/l), and (C4) the Mayo2004-score (1 vs. 2 vs. 3). Significant difference between the groups is depicted by one asterisk (*) for a level of $p < 0.05$, two asterisks (**) for a level of $p < 0.01$, and three (***) for $p < 0.001$ with corresponding patient numbers being depicted in the boxplots.

Supplementary Figure S6. Expression of CD38 based on flow cytometry in malignant plasma cells with regards to clinical parameters.

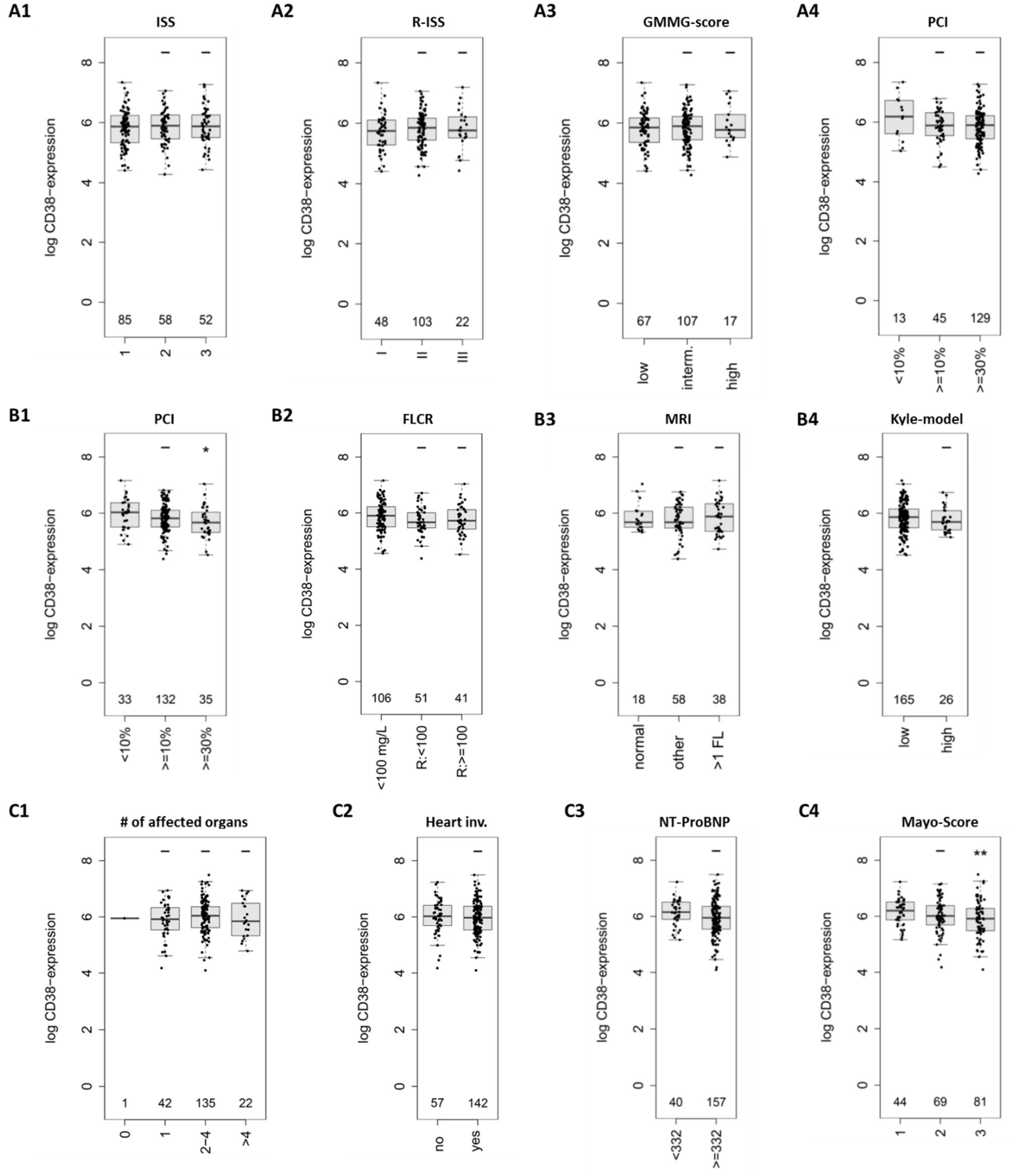

CD38-expression on protein level as assessed by flow cytometry and association with clinical risk factors and surrogates of tumor mass in (**A**) symptomatic myeloma. Shown are (A1) ISS-stage (I vs.

II vs. III), (A2) revised ISS-stage (I vs. II vs. III), (A3) GMMG-score (low vs. intermediate vs. high risk), and (A4) plasma cell infiltration (PCI; <10% vs. ≥10% vs. ≥30%). (**B**) Asymptomatic myeloma. Shown are local and global surrogates of tumor mass, i.e. (B1) plasma cell infiltration (<10% vs. ≥10% vs. ≥30%), (B2) free light chain ratio, (B3) presence of alterations in whole body MRI (wb-MRI), i.e. presence of >1 focal lesion vs. other alterations vs. no alterations (according to (1)), and (B4) tumor mass according to Kyle *et al*. (low vs. high). (**C**) AL-amyloidosis. Shown are organ involvement with regards to (C1) number of affected organs (1 vs. 2-4 vs. >4), (C2) presence of heart involvement, (C3) NT-proBNP level (<332 ng/l vs. ≥332 ng/l), and (C4) the Mayo2004-score (1 vs. 2 vs. 3). Significant difference between the groups is depicted by one asterisk (*) for a level of $p < 0.05$, two asterisks (**) for a level of $p < 0.01$, and three (***) for $p < 0.001$ with corresponding patient numbers being depicted in the boxplots.

Supplementary Figure S7. RSEM transcript analysis (same patients as in Figure 4).

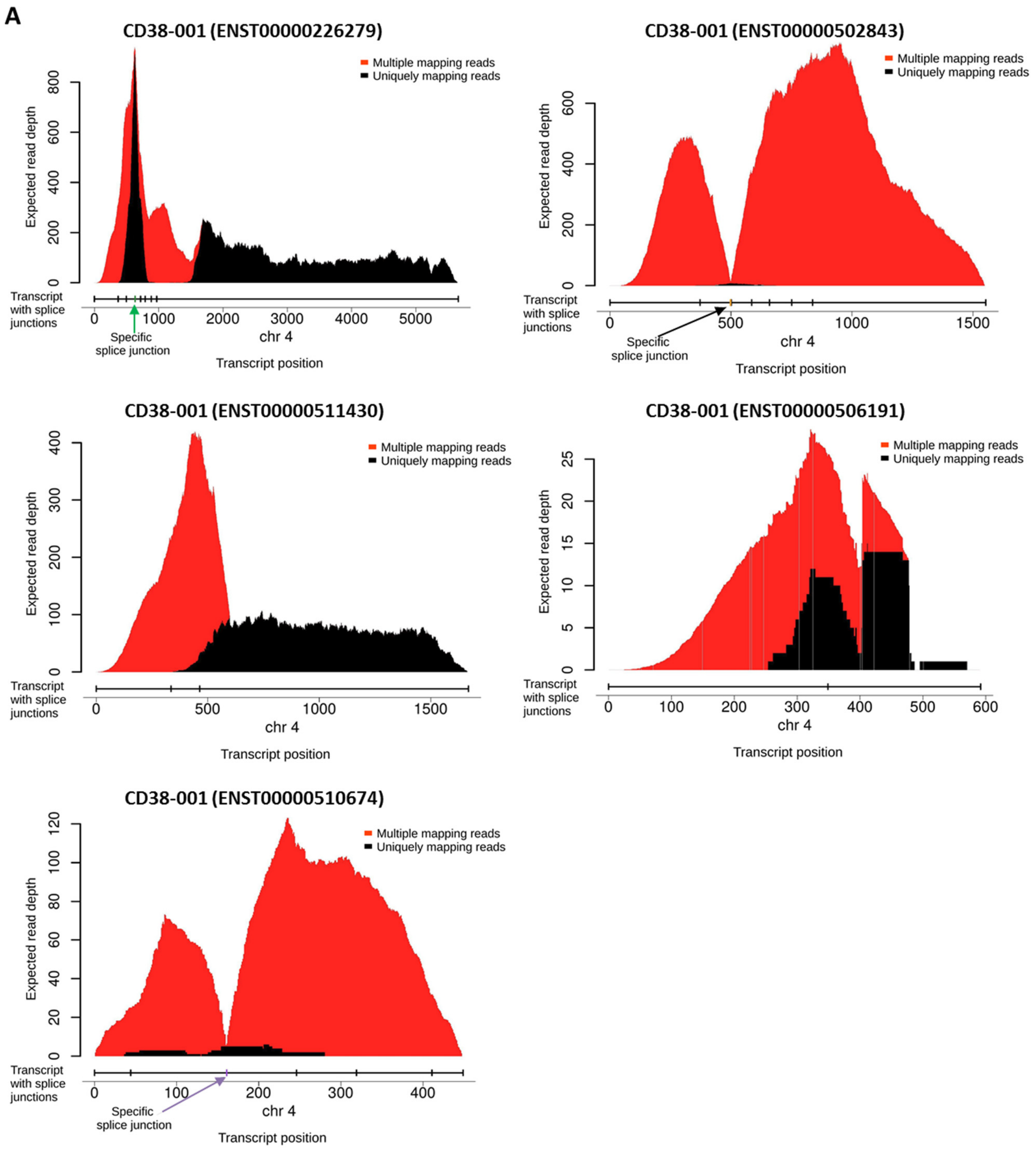

B

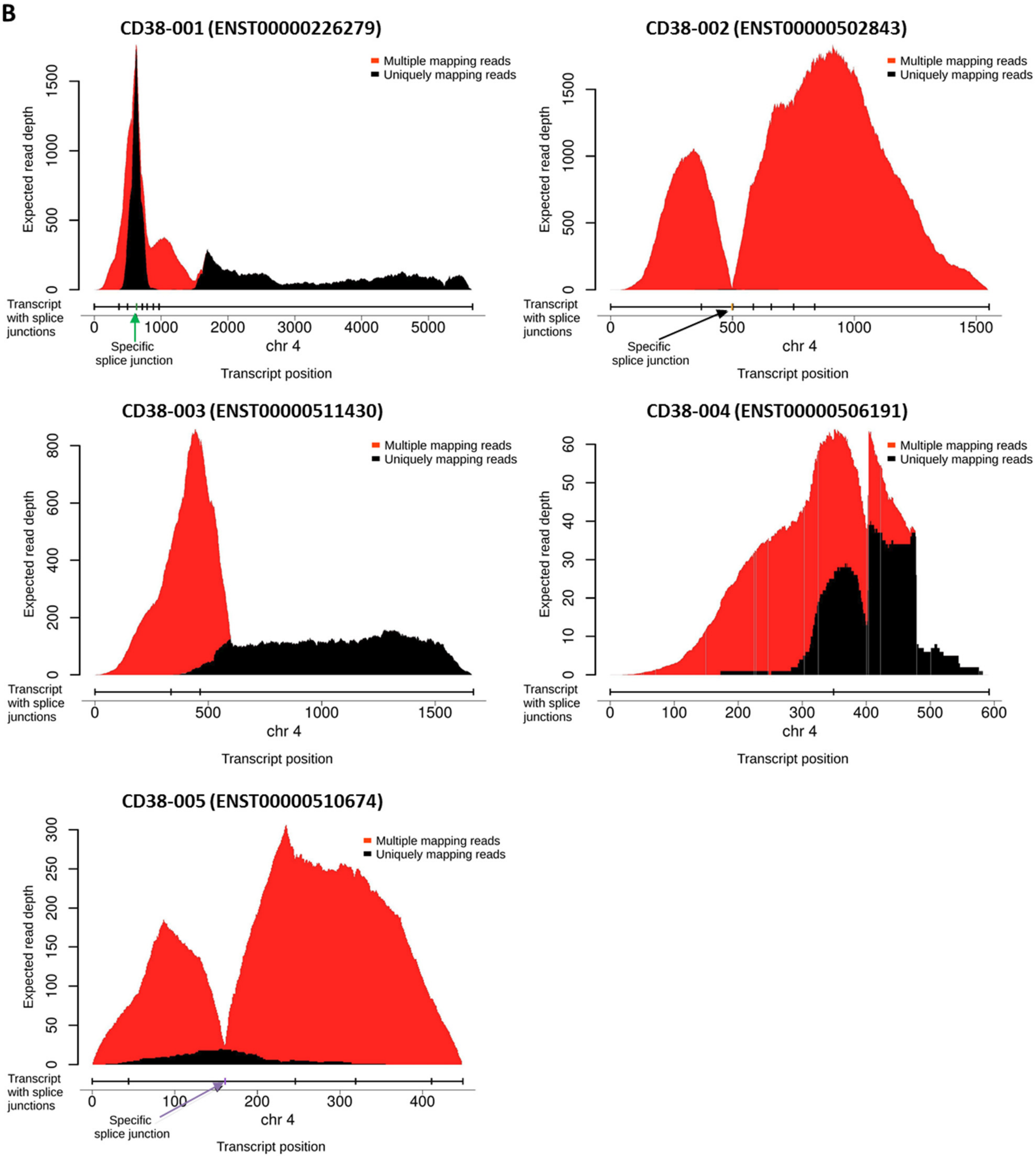

Histograms depicting the read depth for each position of the five transcripts with CD38-001 and CD38-005 being protein coding. Uniquely mapping reads are shown in black, multiple mapping reads in red. Arrows highlight specific splice junctions: One junction exists only in CD38-001, depicted in green. The splice junction specific for CD38-005 is depicted in lilac. (**A**) Exemplary data for a patient

showing expression of CD38-001 only. (**B**) Exemplary patient showing additional alternative splicing in terms of expression of CD38-005 but with low frequency. See also Table 4.

SUPPLEMENTARY TABLES

Supplementary Table S1. Patient characteristics.

Baseline characteristics of patients with available gene expression data (as the largest cohort) are shown. AL, AL-amyloidosis. MGUS, monoclonal gammopathy of unknown significance. AMM, asymptomatic multiple myeloma. MM, multiple myeloma. MMR, relapsed/refractory multiple myeloma. NA, not available. Ten myeloma patients developed secondary AL-amyloidosis during their course of disease.

Variable	Level	All		MGUS		AL		AMM		MM		MMR	
		n	%	n	%	n	%	n	%	n	%	n	%
**Total number of patients**		**1371**		**62**		**196**		**259**		**764**		**90**	
Sex	male	778	56.7	119	60.7	32	51.6	133	51.4	439	57.5	55	61.1
	female	593	43.3	77	39.3	30	48.4	126	48.6	325	42.5	35	38.9
Age [years]	≤60	672	49.	86	43.9	28	45,2	117	45.2	406	53.1	35	38.9
	>60	698	50.9	110	56.1	34	54,8	142	54.8	358	46.9	54	60.0
	NA	1	0.1	0	0.0	0	0.0	0	0.0	0	0.0	1	1.1
Type	IgA	262	19.1	20	10.2			61	23.6	156	20.4	15	16.7
	IgG	769	56.1	54	27.6			181	69.9	452	59.2	57	63.3
	IgD	7	0.5	3	1.5			0	0.0	3	0.4	1	1.1
	Bence Jones	269	19.6	108	55.1			15	5.8	140	18.3	14	15.6
	Double gammopathy	3	0.2	2	1.0			1	0.4	0	0.0	0	0.0
	Asecretory	10	0.7	0	0.0			0	0.0	8	1.0	2	2.2
	Hyposecretory	1	0.1	0	0.0			0	0.0	0	0.0	1	1.1
	Other	2	0.1	0	0.0			1	0.4	1	0.1	0	0.0
Light chain type	Kappa	756	55.1	43	21.9			156	60.2	509	66.6	56	62.2
	Lambda	405	29.5	144	73.5			101	39.0	242	31.7	31	34.4
	Kappa + lambda	2	0.1	1	0.9			2	0.8	0	0.0	0	0.0
	NA	1	0.1	0	0.0			0	0.0	1	0.1	0	0.0
AL type	Kappa					44	22.4						
	Lambda					152	77.6						
Plasma cell infiltration [%]	<10	205	15.0	53	85.5	71	36.2	26	10.0	51	6.7	11	12.2
	≥10	451	32.9	8	12.9	104	53.1	167	64.5	159	20.8	28	31.1
	≥30	343	25.0	0	0.0	16	8.2	48	18.5	265	34.7	21	23.3
	≥60	262	19.1	0	0.0	5	2.6	9	3.5	245	32.1	12	13.3
	NA	110	8.0	1	1.6	0	0.0	9	3.5	44	5.8	18	20.0
ISS stage	1	610	44.5	44	22.4	52	83.9	204	78.8	299	39.1	31	34.4
	2	341	24.9	50	25.5	4	6.5	29	11.2	246	32.2	16	17.8
	3	233	17.0	21	10.7	4	6.5	11	4.2	193	25.3	7	7.8
	NA	187	13.6	81	41.3	2	3.2	15	5.8	26	3.4	36	40.0

Supplementary Table S2. Hazard ratios of CD38-expression (GEP) and chromosomal aberrations as well as gene expression-based factors for patients with symptomatic multiple myeloma in univariate analysis.

GPI, gene expression-based proliferation index.

Name	Variable	Hazard Ratio	Confidence Intervall	*p* value	Events	No.
CD38-expression	low				91	135
	high	0.78	0.6 to 1	0.03	371	568
Del 17p13	no del 17p13				392	610
	del 17p13	1.53	1.2 to 2	0.003	58	80
Gain 1q21	2 copies				251	418
	>2 copies	1.65	1.4 to 2	7.47e-07	165	221
	>3 copies	2.23	1.5 to 3.3	3.16e-05	31	46
t(4;14)	no t(4;14)				388	600
	t(4;14)	1.75	1.4 to 2.3	1.35e-05	72	97
UAMS70-gene score	low risk				325	524
	high risk	1.94	1.6 to 2.4	1.2e-10	137	179
GPI	low risk				154	256
	medium risk	1.4	1.1 to 1.7	0.001	238	363
	high risk	2.42	1.8 to 3.2	1.29e-09	70	84

Supplementary Table S3. Multivariate analysis of CD38-expression (GEP) and chromosomal aberrations regarding event-free survival of patients with symptomatic multiple myeloma.

GEP, gene expression profiling. CI, confidence interval.

Name	Variable	Hazard ratio	Confidence intervall	CI_lower	CI_upper	*p* value	Log Rank *p* value (Scoretest)	No.	R^2	max R^2	concordance	se.concordance
CD38 GEP & t(4;14) & del17p13 & gain1q21 & t(11;14) & hyperdiploidy	CD38 GEP	0.88	0.8 to 1	0.806	0.968	0.008	1,83E-09	615	0.078	0.999	0.616	0.017
	t(4;14)	1.68	1.2 to 2.3	1.202	2.335	0.002						
	del 17p13	1.37	1 to 1.8	1.016	1.850	0.039						
	1q21 >2 copies	1.63	1.3 to 2	1.309	2.037	0.00001						
	1q21 >3 copies	2.02	1.3 to 3	1.340	3.049	0.0008						
	t(11;14)	1.16	0.9 to 1.6	0.853	1.584	0.34						
	Hyperdiploidy	0.98	0.8 to 1.3	0.762	1.255	0.86						

References

1. Hillengass J, Fechtner K, Weber M-A, Bäuerle T, Ayyaz S, Heiss C, et al. Prognostic significance of focal lesions in whole-body magnetic resonance imaging in patients with asymptomatic multiple myeloma. *J Clin Oncol* (2010) 28(9):1606-10. doi: 10.1200/JCO.2009.25.5356.
